# National and Subnational Trends in Ischemic Heart Disease Burden in Iran, 1990–2023: A Systematic Review

**DOI:** 10.1002/clc.70487

**Published:** 2026-10-07

**Authors:** Mehrdad Rabiee Rad, Bahar Darouei, Ghazal Ghasempour Dabaghi, Reza Amani‐Beni, Fahimeh Bagheri, Shahla Shahidi, Noushin Mohammadifard, Fatemeh Sabeti, Sana Azizian, Ali Pourmoghaddas, Shahin Shirani, Mohammadreza Sabri, Nizal Sarrafzadegan

**Affiliations:** ^1^ Interventional Cardiology Research Center, Cardiovascular Research Institute Isfahan University of Medical Sciences Isfahan Iran; ^2^ Isfahan Cardiovascular Research Center, Cardiovascular Research Institute Isfahan University of Medical Sciences Isfahan Iran; ^3^ Heart Failure Research Center, Cardiovascular Research Institute Isfahan University of Medical Sciences Isfahan Iran; ^4^ Department of Cardiology Tehran University of Medical Sciences, Dr Ali Shariati Hospital Tehran Iran; ^5^ Pediatric Cardiovascular Research Center, Cardiovascular Research Institute Isfahan University of Medical Sciences Isfahan Iran; ^6^ Faculty of Medicine, School of Population and Public Health University of British Columbia Vancouver British Columbia Canada

**Keywords:** coronary artery disease, epidemiology, health status disparities, incidence, mortality, prevalence

## Abstract

**Background:**

Ischemic heart disease (IHD) remains a major cause of morbidity and mortality in Iran, but evidence is dispersed across model‐based estimates, population studies, registries, hospital surveillance, and death‐registration data. We synthesized national and subnational evidence on IHD burden in Iran from 1990 to 2023.

**Methods:**

PubMed/MEDLINE and Scopus were searched through August 15, 2026, supplemented by Google Scholar and citation searching. Eligible studies reported incidence, prevalence, mortality, hospitalization, case fatality, post‐myocardial infarction survival, or burden‐of‐disease measures. Model‐based and empirical evidence were synthesized separately, overlapping data sources were mapped, and findings were narratively synthesized because of heterogeneity.

**Results:**

Fifty‐two reports representing 35 evidence‐source families were included. Within GBD 2019, age‐standardized IHD mortality declined from 285.7 to 163.6 per 100 000 between 1990 and 2019, incidence from 898.3 to 829.1, and prevalence from 6250.6 to 6198.5 per 100 000, while incident cases increased from 218 181 to 593 001. Successive GBD vintages also showed declining age‐standardized mortality but were not directly comparable because of modeling differences. Empirical studies generally showed higher coronary event rates in men, while Isfahan surveillance and the Iranian MI Registry indicated higher short‐term post‐MI case fatality among women. Burden increased with age, provincial variation was substantial, and urban‐rural patterns were inconsistent. Hospital surveillance suggested improving short‐term outcomes.

**Conclusions:**

Successive GBD analyses estimated substantial declines in age‐standardized IHD mortality, while absolute burden remained large and differences persisted by age, sex, geography, and data source. Stronger prevention and geographically representative surveillance are needed.

## Graphical abstract

In Iran, age‐standardized ischemic heart disease mortality and incidence declined from 1990 to 2019, while absolute incident cases increased substantially. Burden remained higher in men and older adults, with marked provincial variation, highlighting the need for stronger prevention and more standardized national surveillance.

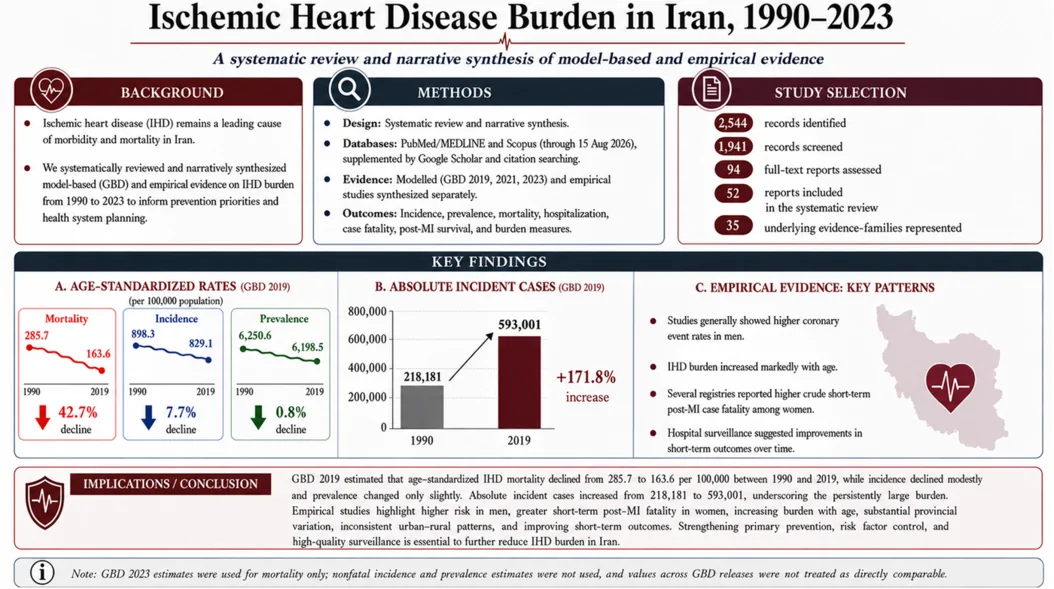

## 1 Introduction

Ischemic heart disease (IHD) remains a leading global cause of morbidity and mortality, with approximately 315 million prevalent cases and 9.24 million deaths worldwide in 2022 [1]. The burden is particularly substantial in the Eastern Mediterranean Region (EMR), where age‐standardized IHD prevalence increased from 5.0% to 5.5% between 1990 and 2019 despite a declining global trend [2]. Although IHD mortality and DALY rates in the region declined by 11.4% and 15.4%, respectively, both remained higher than the corresponding global rates [2]. In 2019, IHD accounted for approximately 0.8 million deaths and 18.0 million DALYs across the Middle East and North Africa [3].

The burden of IHD varies across regions because of differences in the stage of epidemiologic transition, cardiovascular risk‐factor profiles, socioeconomic conditions, and healthcare systems [2, 4]. Given these disparities, evaluating IHD burden at a country‐specific level is essential, particularly in countries such as Iran, where demographic, epidemiological, and health‐system characteristics may shape disease burden [5, 6].

In Iran, evidence on IHD burden is distributed across successive GBD estimates, national and regional registries, population‐based cohorts, hospital surveillance systems, and death‐registration studies [7, 8, 9]. These sources differ substantially in disease definitions, denominators, geographic coverage, and outcome ascertainment, while several publications derive from overlapping underlying populations or model vintages [10, 11, 12]. A systematic synthesis that distinguishes model‐based estimates from empirical evidence and separates population incidence and mortality from hospitalization and case‐fatality measures is therefore needed. Accordingly, this systematic review aimed to characterize national and subnational patterns and temporal trends in IHD incidence, prevalence, hospitalization, mortality, and related burden measures in Iran from 1990 to 2023, with particular attention to variation by sex, age, and geography.

## 2 Methods

### 2.1 Study Design and Reporting

This systematic review and narrative synthesis evaluated national and subnational evidence on ischemic heart disease burden in Iran from 1990 to 2023. The review was conducted and reported according to the PRISMA 2020 statement [13] and was registered in PROSPERO (CRD420261479564).

### 2.2 Eligibility Criteria

Studies were eligible if they reported quantitative estimates of IHD burden in populations from Iran at the national, provincial, regional, or community level. Eligible disease constructs included IHD, coronary heart disease (CHD), coronary artery disease (CAD), and myocardial infarction (MI). Studies of acute coronary syndrome (ACS), ST‐segment elevation myocardial infarction (STEMI), non‐ST‐segment elevation myocardial infarction (NSTEMI), or unstable angina were eligible when they contributed a prespecified population, registry, or surveillance measure, such as hospitalization, temporal or geographic burden, case fatality, or post‐event survival. The primary outcomes were population‐based incidence, prevalence, and mortality. Secondary outcomes included hospitalization or admission rates, case‐fatality measures, short‐ or longer‐term survival after MI, and burden measures including disability‐adjusted life years (DALYs), years of life lost (YLLs), and years lived with disability (YLDs).

Eligible evidence sources included population‐based cross‐sectional surveys, prospective population cohorts, national and regional registries, death‐registration studies, hospital‐based surveillance systems reporting informative temporal or geographic burden estimates, and national or subnational burden‐of‐disease or statistical modeling studies. Hospital‐based studies without a population denominator were eligible when they evaluated an explicit temporal or geographic surveillance objective, including longitudinal admission or case‐fatality trends, registry‐based geographic comparisons, or multicenter comparisons of major health‐system disruptions. Studies limited to clinical characteristics, individual prognostic factors, or treatment and procedural outcomes without an explicit surveillance objective were excluded.

Studies restricted primarily to risk‐factor associations, treatment effectiveness, selected clinical prognostic cohorts, angiographic convenience samples, or composite cardiovascular outcomes from which an IHD‐specific estimate could not be separated were excluded. Reviews, protocols, editorials, letters without original quantitative data, case reports, and duplicate or redundant reports without a unique eligible contribution were also excluded. No restrictions were imposed according to publication language or publication year. Full eligibility criteria are presented in Table S1.

### 2.3 Information Sources and Search Strategy

PubMed/MEDLINE and Scopus were systematically searched from database inception through 15 August 2026. Search strategies combined controlled vocabulary and free‐text terms for IHD, CHD, CAD, MI, acute coronary syndrome, STEMI, NSTEMI, and unstable angina with terms for Iran and for incidence, prevalence, mortality, epidemiology, burden, temporal trends, and case fatality. The supplementary Google Scholar search additionally incorporated terms related to hospitalization, admission, registries, surveillance, and survival. No language or publication‐type restrictions were applied. Google Scholar was used as a supplementary source, with the first 200 results ranked by relevance screened. Reference lists and citation links of potentially relevant reports were also examined to identify additional eligible publications. The complete database‐specific search strategies are provided in Supporting Information S1: Table 2.

### 2.4 Study Selection and Data Extraction

Two reviewers (M.R.R. and G.G.D) independently screened all titles and abstracts against the predefined eligibility criteria. Full texts of potentially eligible reports were subsequently assessed independently by the two reviewers, and all final inclusion and exclusion decisions were verified before the evidence set was finalized. Disagreements were resolved by consensus, with consultation of a third reviewer (N.S.) when necessary. Reasons for exclusion at the full‐text stage were documented and are presented in Supporting Information S1: Table 3. Before finalization, eligibility decisions were re‐audited with particular attention to publications derived from overlapping cohorts, registries, surveillance systems, and burden‐of‐disease datasets. Reports that could not be retrieved were recorded separately and were not classified as eligible or ineligible solely on the basis of their abstracts.

For each included report, data were extracted on author and publication year, study period, geographic setting, study design and data source, population and sample size, disease definition and ascertainment method, denominator, incidence and prevalence estimates, hospitalization measures, mortality, case fatality or survival, DALYs, YLLs and YLDs, age‐ and sex‐specific findings, geographic or urban‐rural comparisons, measures of uncertainty, and relevant methodological limitations. Numerical estimates were retained in the units and epidemiological form reported by the original studies. Population incidence, prevalence, hospital admission rates, case fatality, post‐MI survival, and population mortality were treated as distinct epidemiological measures and were not considered interchangeable. Two reviewers (B.D. and R.A.B.) independently extracted data from the included reports, with disagreements resolved by consensus and consultation with a third reviewer (N.S.) when necessary.

### 2.5 Management of Overlapping Populations and Data Sources

Multiple publications were identified from several common underlying data sources, including the Tehran Lipid and Glucose Study, the Isfahan Healthy Heart Program and Isfahan Cohort Study, the Isfahan AMI/ACS surveillance network, the Iranian Myocardial Infarction Registry, and the Yazd Healthy Heart Project. Publications derived from the same underlying population or registry were mapped explicitly before synthesis. Overlapping reports were retained when they provided a distinct eligible outcome, study period, subgroup, or geographic analysis. Reports that did not provide a unique contribution beyond a more complete publication from the same source were excluded as redundant. Overlapping reports were therefore not interpreted as statistically independent studies. The relationship between overlapping reports and their underlying data sources is detailed in Supporting Information S1: Table 4.

The same principle was applied to Global Burden of Disease (GBD) publications. Analyses using the same GBD release were treated as derivative reports from a common modeling framework rather than independent epidemiological datasets. Different GBD releases were treated as separate model vintages because successive releases may incorporate changes in source data, disease definitions, statistical models, redistribution procedures, and standard populations. Consequently, differences between estimates from different GBD releases were not interpreted as direct temporal changes in disease burden.

### 2.6 Methodological Appraisal

Because the included evidence comprised heterogeneous study designs, methodological appraisal was performed using design‐specific approaches rather than a single quality score. Population‐based prevalence studies were assessed using the Joanna Briggs Institute (JBI) Checklist for Prevalence Studies, cohort studies using the JBI Cohort Studies tool, registry and hospital‐surveillance reports primarily representing defined case series using the JBI Case Series checklist, and non‐randomized policy evaluations using the JBI Quasi‐Experimental Studies tool [14, 15, 16, 17]. For registry and hospital‐surveillance studies, the applicable JBI checklist was supplemented by prespecified surveillance‐specific domains addressing source‐population and catchment coverage, case ascertainment, completeness of event capture and follow‐up, appropriateness of the denominator, and temporal or geographic consistency of the reported estimates. Population mortality studies without a directly corresponding JBI checklist were evaluated using prespecified domains covering completeness of death registration, cause‐of‐death classification, denominator validity, standardization, and temporal consistency. Model‐based and burden‐of‐disease studies were assessed using domains informed by the Guidelines for Accurate and Transparent Health Estimates Reporting (GATHER), including transparency of input data, estimation methods, uncertainty, and model dependence [18]. GATHER was used as a reporting and transparency framework rather than a risk‐of‐bias instrument.

Methodological appraisal was performed independently by two reviewers (B.D. and R.A.B.). Disagreements in individual domain assessments or overall judgments were resolved by discussion and consensus, with consultation of a third reviewer (N.S.) when necessary. Overall judgments were assigned after consideration of the pattern and importance of domain‐level concerns and were not based on a numerical score or exclusion threshold. The categories “No major methodological concern,” “Some methodological concerns,” and “Major concern for specific estimate” were review‐level summary judgments developed for this synthesis rather than validated JBI or GATHER categories. Appraisal findings were used to inform interpretation of the evidence and were not used as a basis for study exclusion.

### 2.7 Data Synthesis

Substantial heterogeneity was anticipated and observed across study populations, case definitions, ascertainment methods, denominators, study designs, follow‐up periods, geographic coverage, and reported measures. Therefore, quantitative meta‐analysis was not considered appropriate, and findings were synthesized narratively. Evidence was first separated into model‐based/burden‐of‐disease estimates and empirical studies. Empirical evidence was further organized into population‐based incidence and prevalence studies, registry‐ and hospital‐based surveillance studies, and population‐based mortality studies. Findings were summarized according to time period, sex, age, and geography where corresponding data were available. Absolute numbers and age‐standardized rates were considered separately. Hospital admission or hospitalization‐derived rates were not interpreted as population incidence, and in‐hospital or 28‐day case fatality was not interpreted as population mortality. For GBD estimates, 95% uncertainty intervals were reported where available and trends were preferentially interpreted within the same GBD model vintage rather than by comparing absolute values across successive GBD releases.

## 3 Results

### 3.1 Study Selection

The database search identified 2544 records, of which 603 duplicates were removed, leaving 1941 unique records for title and abstract screening. Of these, 78 reports were sought for full‐text retrieval, and were assessed for eligibility. Supplementary searching through Google Scholar and citation tracking identified 16 additional reports for full‐text assessment. Overall, 94 full‐text reports were assessed, of which 42 were excluded for prespecified reasons, leaving 52 reports in the final systematic review and narrative synthesis (Figure 1; Supporting Information S1: Table 3) [3, 7, 8, 9, 10, 11, 12, 19, 20, 21, 22, 23, 24, 25, 26, 27, 28, 29, 30, 31, 32, 33, 34, 35, 36, 37, 38, 39, 40, 41, 42, 43, 44, 45, 46, 47, 48, 49, 50, 51, 52, 53, 54, 55, 56, 57, 58, 59, 60, 61, 62, 63].

**FIGURE 1:**
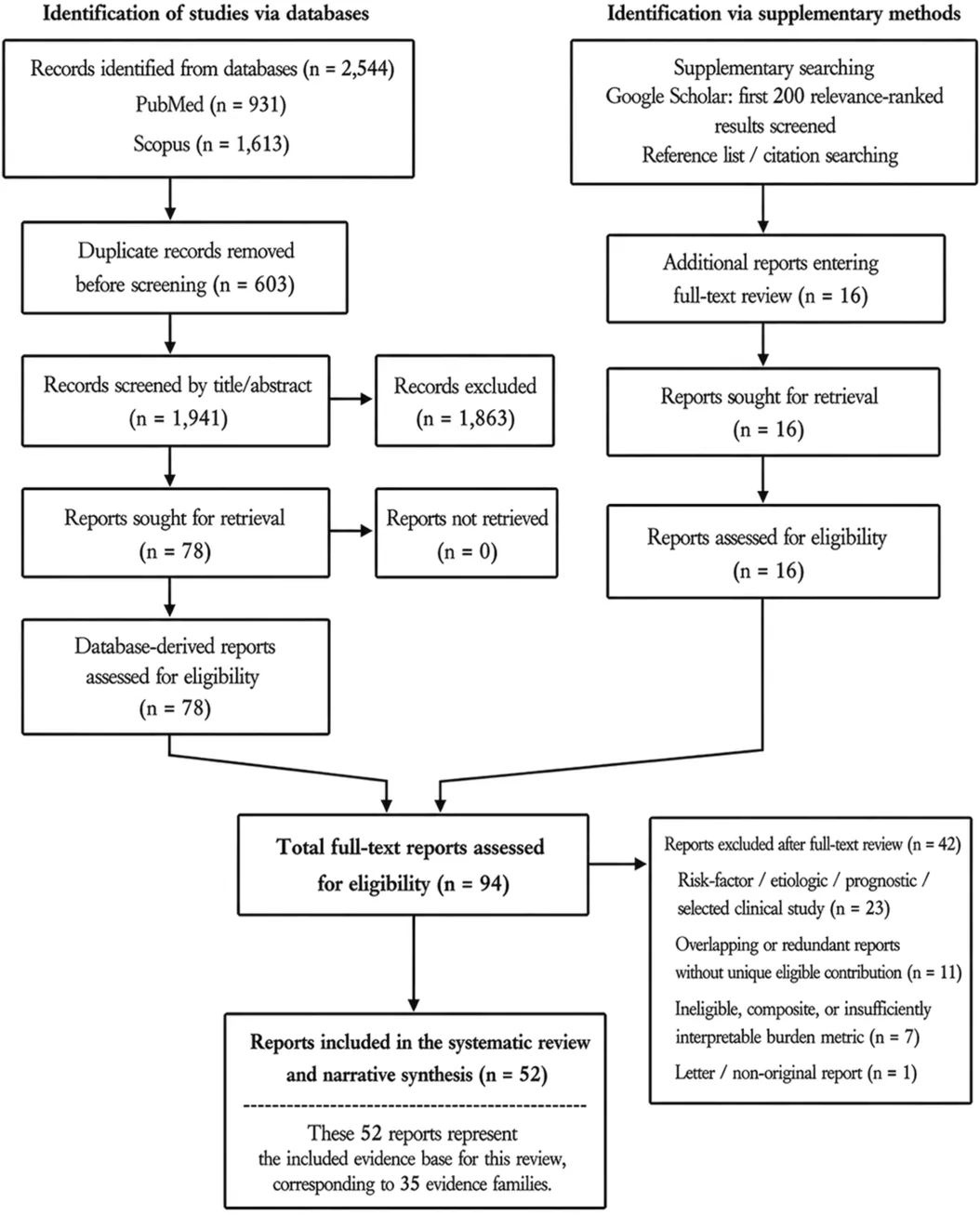
PRISMA 2020 flow diagram.

Several included reports originated from the same underlying cohorts, registries, surveillance systems, or GBD model vintages. These reports were retained only when they contributed distinct eligible outcomes, time periods, subgroups, or geographic information and were not treated as independent evidence sources in the synthesis. The principal overlaps involved the Tehran Lipid and Glucose Study, the Isfahan Healthy Heart Program/Isfahan Cohort Study, the Isfahan AMI/ACS surveillance network, the Iranian Myocardial Infarction Registry, and the GBD 2019 model family (Supporting Information S1: Table 4).

### 3.2 Characteristics of the Included Evidence

The 52 included reports comprised 13 model‐based or burden‐of‐disease reports and 39 empirical reports. The empirical evidence comprised 18 population‐based incidence or prevalence reports, 17 registry‐ or hospital‐surveillance reports, and four population‐based mortality reports. After accounting for repeated publications from the same cohorts, registries, surveillance systems, and GBD releases, the 52 reports mapped to 35 underlying evidence‐source families rather than 52 independent populations (Tables 1 and 2). Empirical studies included nationally representative surveys, population‐based cohorts, regional community surveys, national and provincial MI registries, multicenter hospital‐surveillance systems, and death‐registration studies. Geographic coverage ranged from national analyses to studies conducted in Tehran, Isfahan and central Iran, the Persian Gulf region, Qazvin, Borujerd, northern Iran, Fars, Chaharmahal and Bakhtiari, Mashhad, Yazd, Hormozgan, Zanjan, Bushehr, and Mazandaran. Model‐based evidence extended back to 1990, whereas most directly observed empirical data were collected from the early 2000s onward.

Disease definitions and ascertainment methods varied substantially across studies. Population‐based reports used combinations of Minnesota‐coded ECG findings, Rose questionnaire criteria, self‐reported physician diagnosis, medical‐record confirmation, and adjudicated clinical outcomes, whereas registry and surveillance studies primarily used clinical, WHO‐MONICA, or ICD‐based definitions. Model‐based studies relied on study‐specific burden frameworks or GBD cause definitions. Accordingly, estimates labeled as IHD, CHD, CAD, MI, ACS, hospitalization, case fatality, and population mortality represented distinct epidemiological constructs and were not treated as interchangeable (Supporting Information S1: Table 5).

### 3.3 Methodological Appraisal

Methodological quality varied across the included evidence. Population‐based prospective cohorts with standardized or adjudicated outcomes generally had fewer concerns, whereas other studies were limited by self‐reported disease, heterogeneous ECG‐ or questionnaire‐based definitions, restricted geographic or age coverage, and attrition in some cohorts. Registry and hospital‐surveillance studies were useful for temporal, geographic, and case‐fatality patterns but were limited for population‐level inference because they captured patients reaching participating hospitals. Hospitalization‐derived rates were therefore not interpreted as population incidence, and in‐hospital or 28‐day case fatality was not interpreted as population mortality. Particular caution was required for the overall rate reported by Namayande et al. because of internal inconsistency with the sex‐specific estimates [47]. Model‐based studies were limited by source‐data quality, correction procedures, and model assumptions. The projected estimates from Kiani et al. were considered particularly assumption‐dependent [35], while the nonfatal GBD 2021 estimates reported by Heidari‐Foroozan et al. were not used because the incidence and prevalence values appeared internally transposed [32]. No study was excluded solely on the basis of methodological appraisal (Supporting Information S1: Table 6).

### 3.4 Model‐Based Estimates of IHD Burden

#### 3.4.1 National Temporal Trends

Across successive GBD vintages, age‐standardized IHD mortality declined substantially within each model series, although the magnitude differed across releases (Table 1). In GBD 2010, the age‐standardized mortality rate decreased from 222 per 100 000 (95% UI: 207, 243) in 1990 to 175 per 100 000 (95% UI: 152, 190) in 2010, while the absolute number of deaths increased from 52 874 to 89 250 [37]. In GBD 2019, age‐standardized mortality declined from 285.7 per 100 000 (95% UI: 260.8, 307.1) in 1990 to 163.6 per 100 000 (95% UI: 149.0, 176.2) in 2019, and the DALY rate decreased from 5437.6 to 2842.7 per 100 000 [8]. GBD 2021 similarly estimated a decline in age‐standardized mortality from 259.88 per 100 000 (95% UI: 238.06, 276.23) in 1990 to 146.11 per 100 000 (95% UI: 130.71, 156.95) in 2021, accompanied by a reduction in the DALY rate from 5166.82 to 2731.30 per 100 000 [32]. The GBD 2023 cause‐of‐death analysis estimated an age‐standardized IHD mortality rate of 127.2 per 100 000 (95% UI: 104.5, 149.1) in 2023 [58]. Because successive GBD releases use updated data sources and modeling procedures, these estimates were interpreted within each model vintage rather than as a single continuous observed series.

**TABLE 1:** Model‐based and burden‐of‐disease estimates of ischemic heart disease in Iran.

Evidence source/family	Included report(s)	Period/geographic coverage	Approach	Incidence or prevalence	Mortality/burden	Interpretation/overlap
**National burden models and GBD estimates**						
Iran National Burden of Disease 2003	Naghavi et al., 2009 [1]	2003; national estimates and six selected provinces	WHO/NBD burden modeling	Incidence/prevalence NR	IHD mortality 109/100 000; YLL 771/100 000; YLD 539/100 000; DALY 1310/100 000; total IHD DALYs 871 336	Historical national burden model; not directly comparable with later GBD vintages.
GBD 2010	Maracy et al., 2015 [2]	1990–2010; Iran	GBD 2010 modeled IHD burden	Incidence/prevalence NR	AS mortality 222 (207–243) → 175 (152–190)/100 000; DALY ASR 4720 → 3245; deaths 52 874 → 89 250	Age‐standardized mortality fell while absolute deaths increased; release‐specific model.
GBD 2019 model family	Koolaji et al., 2023; Mousavi et al., 2023; Karamitanha et al., 2024; Aminorroaya et al., 2024 [3, 4, 5, 6]	1990–2019; national, 31 provinces; age ≥ 70 subgroup	GBD 2019 national/subnational and age‐specific analyses	AS incidence 898.3 (784.0–1,026.5) → 829.1 (719.9–945.2)/100,000; AS prevalence 6,250.6 (5678.7–6852.2) → 6198.5 (5644.4–6814.6)/100 000	AS mortality 285.7 (260.8–307.1) → 163.6 (149.0–176.2)/100 000; DALY ASR 5437.6 → 2842.7. In 2019, highest provincial incidence was reported in North Khorasan and highest mortality in Golestan; among adults ≥ 70, prevalence 52886.4/100 000 and mortality 1932.9/100 000	The four reports share the same GBD 2019 model vintage and are not independent evidence.
GBD 2021	Heidari‐Foroozan et al., 2025 [7]	1990–2021; national/subnational Iran	GBD 2021 IHD/CVD burden analysis	Nonfatal IHD estimates not tabulated because incidence/prevalence labels and values appear internally transposed in the article	AS IHD mortality 259.88 (238.06–276.23) → 146.11 (130.71–156.95)/100 000; DALY ASR 5166.82 → 2731.30	Mortality/DALY estimates retained; incidence/prevalence withheld for data‐integrity reasons.
GBD 2023	Sepanlou et al., 2026 [8]	1990–2023; national and all 31 provinces	GBD 2023 cause‐specific mortality and life‐expectancy decomposition	Incidence/prevalence NR in this report	2023 IHD AS mortality 127.2 (104.5–149.1)/100 000; provincial ASMR 88.1 (67.2–108.3) in Tehran to 262.8 (213.4–310.5) in Qom; mortality decline contributed 2.61 y to national life‐expectancy gain	Mortality‐focused report; does not support national 2023 IHD incidence/prevalence values.
**Other national and subnational burden/mortality models**						
Isfahan burden model	Shams‐Beyranvand et al., 2017 [9]	2014; Isfahan Province	Regional burden modeling using mortality/disability inputs	NR	DALYs 43517.71; YLL 31891.79; YLD 11625.92; DALY rate 3953.31 (3471.33‐4431.25)/100 000; men 4412.33, women 3476.66	Subnational modeled burden; dependent on completeness and correction assumptions.
West Azerbaijan MI burden	Ebrahimi et al., 2017 [10]	2012–2013; West Azerbaijan	Regional MI burden calculation	Reported MI prevalence 376/100 000	MI accounted for 18.8% of all deaths; DALYs 53 804 y (17.7/1,000), YLL 52 170, YLD 1634; 7235 MI occurrences	Regional MI‐specific burden; event/death capture completeness matters.
Isfahan mortality model	Kiani et al., 2019 [11]	Observed 2004–2013; projected 2014–2018; Isfahan	Age‐period‐cohort/MIAMOD modeling	NR	During observed 2004–2013, age‐adjusted IHD mortality increased on average 13.6% in men and 14.7% in women; projected annual increases ~14.1% and 10%, respectively	Projection‐dependent estimates; not directly comparable with corrected DRS or GBD series.
Kurdistan burden study	Moradi et al., 2021 [12]	2011–2017; Kurdistan Province	Regional CVD burden calculation	NR	IHD accounted for 71102.3 DALYs (33.8% of total CVD DALYs)	Subnational burden estimate; depends on source‐data completeness.
Corrected Iranian DRS mortality model	Naderimagham et al., 2022 [13]	1990–2015; national/provincial	Corrected death‐registration data + spatiotemporal/Gaussian‐process modeling	NR	AS IHD mortality 49.0 (34.8–67.9) in 1990 → peak 146.5 (112.0‐187.4) in 2003 → 91.6 (71.9–113.7) in 2015/100 000; 2015 women 79.1, men 104.1; provincial range 58.0–136.2	Modeled correction for incomplete/misclassified death registration.

*Note*: Values in parentheses are 95% uncertainty intervals for GBD/model estimates unless otherwise stated. Successive GBD rounds are separate model vintages and should not be interpreted as one directly comparable observed time series.

Abbreviations: AS, age‐standardized; ASR, age‐standardized rate; DALY, disability‐adjusted life year; DRS, death registration system; GBD, Global Burden of Disease; IHD, ischemic heart disease; NBD, National Burden of Disease; NR, not reported; YLD, years lived with disability; YLL, years of life lost.

For nonfatal burden, GBD 2019 estimated that the age‐standardized IHD incidence rate decreased from 898.3 per 100 000 (95% UI: 784.0, 1026.5) in 1990 to 829.1 per 100 000 (95% UI: 719.9, 945.2) in 2019, while the age‐standardized prevalence rate changed only slightly, from 6250.6 per 100 000 (95% UI: 5678.7, 6852.2) to 6198.5 per 100 000 (95% UI: 5644.4, 6814.6) [8]. In contrast to these age‐standardized trends, the absolute number of incident cases increased from 218 181 to 593 001 and prevalent cases from approximately 1.49 million to 4.34 million over the same period. Exact nonfatal estimates from the GBD 2021 report were not included because the published incidence and prevalence labels and values appeared internally transposed. The retained GBD 2023 report was mortality‐focused and did not provide national IHD incidence or prevalence estimates. Earlier and non‐GBD burden studies provided additional context. The Iran National Burden of Disease study estimated an IHD mortality rate of 109 per 100 000 and a DALY rate of 1310 per 100 000 in 2003, corresponding to 871 336 total IHD DALYs [46]. Regional burden studies also documented substantial disability and premature mortality, including 43517.71 IHD DALYs in Isfahan in 2014 and 71102.3 IHD DALYs in Kurdistan during 2011–2017 [41, 59]. These estimates were not considered directly comparable with GBD estimates because their source data and modeling approaches differed.

#### 3.4.2 Sex, Age, and Subnational Patterns

GBD 2019 showed higher age‐standardized IHD burden in men than in women. In 2019, the age‐standardized incidence rate was 1007.8 per 100 000 in men compared with 652.0 per 100 000 in women, the prevalence rate was 7597.1 versus 4815.8 per 100 000, and the mortality rate was 177.2 versus 150.9 per 100 000, respectively [8]. Among adults aged ≥ 70 years, the GBD 2019 analysis estimated an IHD prevalence of 52886.4 per 100 000 (95% UI: 47543.0, 58381.6) and a mortality rate of 1932.9 per 100 000 (95% UI: 1720.8, 2094.2) in 2019, demonstrating the substantially greater burden at older ages [42].

Subnational estimates also showed considerable geographic variation. Within the GBD 2019 model family, North Khorasan had the highest provincial age‐standardized incidence rate in 2019, whereas Golestan had the highest mortality rate [11]. In the GBD 2023 mortality analysis, provincial age‐standardized IHD mortality ranged from 88.1 per 100 000 (95% UI: 67.2, 108.3) in Tehran to 262.8 per 100 000 (95% UI: 213.4, 310.5) in Qom [58]. A separate analysis using corrected Iranian death‐registration data also demonstrated substantial temporal and provincial variation, with age‐standardized IHD mortality increasing from 49.0 per 100 000 (95% UI: 34.8, 67.9) in 1990 to a peak of 146.5 per 100 000 (95% UI: 112.0, 187.4) in 2003 before declining to 91.6 per 100 000 (95% UI: 71.9, 113.7) in 2015 [45].

### 3.5 Empirical Population‐Based Incidence and Prevalence

#### 3.5.1 Incidence

Prospective population‐based cohorts consistently showed higher coronary event rates in men than in women, although absolute estimates varied by phenotype and study population (Table 2; Supporting Information S1: Table 7). In the Isfahan Cohort Study, 219 incident IHD events occurred over 24,379 person‐years, corresponding to an incidence rate of 8.9 per 1000 person‐years (95% CI: 7.8, 10.2); rates were 10.6 per 1000 person‐years (95% CI: 8.8, 12.5) in men and 7.4 per 1000 person‐years (95% CI: 6.0, 9.0) in women [9]. A subsequent analysis from the same cohort reported AMI incidence rates of 352 per 100 000 person‐years in men and 186 per 100 000 person‐years in women, while unstable angina was more frequent in women than men (584 vs. 352 per 100 000 person‐years) [62]. Because these reports arose from the same research program but evaluated different coronary outcomes, they were treated as complementary rather than independent estimates.

**TABLE 2:** Characteristics and principal findings of empirical studies reporting ischemic heart disease burden in Iran.

Evidence source/family	Included report(s)	Period/setting	Design/data source	Phenotype/ascertainment	Principal burden finding(s)	Interpretation/overlap
**Population‐based incidence and prevalence evidence**						
Tehran Lipid and Glucose Study (TLGS)	Ghanbarian et al., 2004; Hadaegh et al., 2009; Khalili et al., 2014; Moazzeni et al., 2020 [14, 15, 16, 17]	Tehran; baseline surveys and follow‐up through 2010/2011	Population‐based cross‐sectional and prospective cohort analyses	ECG‐/Rose‐/self‐report‐defined MI/CHD in early reports; later adjudicated CHD	ECG MI 1.2% overall (2004 report); age‐adjusted CHD prevalence 21.8% (2009 report); CHD incidence 11.9/1000 PY men versus 6.5 women (2014); age‐standardized CHD prevalence 7.71% (7.18–8.24) in 2008–2011	Same cohort framework but different waves, definitions, and outcomes; reports are complementary, not independent populations.
Isfahan Healthy Heart Program/Isfahan Cohort Study	Sadeghi et al., 2006; Sarrafzadegan et al., 2011; Talaei et al., 2013 [18, 19, 20]	Isfahan, Najafabad, Arak; baseline and prospective follow‐up	Population survey and prospective cohort	Rose + Minnesota ECG for baseline CAD; adjudicated IHD/AMI/UAP/SCD in follow‐up	Baseline CAD: women 37.5%, men 22.2%; incident IHD 8.9 (7.8–10.2)/1000 PY; AMI incidence 352/100 000 PY men versus 186 women	Different phenotypes and time windows from the same central‐Iran research program.
Persian Gulf Healthy Heart Study	Nabipour et al., 2006 [21]	Three northern Persian Gulf ports; *n* = 3723 adults ≥ 25 y	Population‐based cluster‐random cross‐sectional study	Minnesota‐coded ECG nonfatal IHD	ECG‐IHD prevalence 12.7% overall; 10.4% men; 14.7% women	Regional ECG‐defined prevalence; not equivalent to physician‐diagnosed IHD.
Qazvin population study	Fakhrzadeh et al., 2008 [22]	Qazvin; *n* = 846 adults ≥ 25 y	Population‐based cross‐sectional study	Possible MI, ischemic ECG changes, angina	Age‐adjusted prevalence: possible MI 4.2%; ischemic ECG changes 36.8%; angina 2.2%	Distinct outcomes should not be summed into a single CHD prevalence.
SuRFNCD‐2011	Abbasi et al., 2018 [23]	National survey; *n* = 11 867	National population survey	Self‐reported CHD and chronic stable angina	CHD 5.3% (4.6–5.9); stable angina 7.7% (4.6–8.7); CHD urban 5.6% versus rural 4.3%	Nationally representative but self‐reported outcomes.
Borujerd population study	Maleki et al., 2019 [24]	Borujerd; *n* = 801 adults > 35 y	Population‐based cross‐sectional study	Rose questionnaire + Minnesota ECG + documented CAD	Probable CAD 18.7%; definite CAD 15.7%	Regional estimate; final diagnostic table values used because abstract wording is inconsistent.
Tabari Cohort	Ghaemian et al., 2020 [25]	Sari/northern Iran; *n* = 10 255 aged 35–70 y	Population‐based cohort baseline	Self‐reported CHD	CHD prevalence 9.2%; rural 12.1% versus urban 7.9%	Regional baseline prevalence; self‐report.
Pars Cohort	Adib et al., 2020 [26]	Fars Province; *n* = 9248 aged 40–75 y	Population‐based cohort baseline	Self‐reported IHD	IHD prevalence 10.2% (9.5–10.9)	Regional baseline prevalence; self‐report.
Shahrekord PERSIAN Cohort	Ahmadi et al., 2021 [27]	Chaharmahal and Bakhtiari; *n* = 10 075 aged 35–70 y	Population‐based cohort baseline	Self‐reported physician‐diagnosed IHD/MI	IHD 5.7%; MI 1.3%; IHD 6.9% men versus 4.7% women; 6.3% urban versus 4.4% rural	IHD and MI retained as separate outcomes.
MASHAD Cohort	Sadabadi et al., 2022 [28]	Mashhad; 6‐y follow‐up	Prospective population cohort	Incident adjudicated CHD	CHD incidence density 19.20 (8.10–30.30)/1000 PY men and 11.60 (7.30–15.90) women	Regional prospective incidence evidence.
Yazd Healthy Heart Project	Sarebanhassanabadi et al., 2024 [29]	Yazd; baseline 2005–2006; mean follow‐up 9.9 y; *n* = 1552 analyzed	Community‐based prospective cohort	Incident clinical CAD	225 incident CAD events; cumulative incidence 14.5% (12.4–16.6); incidence 16.8 men versus 12.0 women/1,000 PY	~20% attrition from 2,000 recruited; 2025 etiological report using the same events was excluded as redundant.
Hormozgan population survey	Farshidi et al., 2025 [30]	Hormozgan; Jun‐Dec 2023; *n* = 6289 adults ≥ 20 y	Multistage population‐based cross‐sectional survey	Self‐report with medical‐record review/cardiologist confirmation	IHD prevalence 6.7%; men 7.5%, women 6.1%; urban 6.9%, rural 6.5%	Strong recent subnational prevalence estimate.
Iran STEPS 2021	Sabbagh Alvani et al., 2026 [31]	National; *n* = 16 552 adults ≥ 40 y	Nationally representative cross‐sectional analysis	Self‐reported physician‐diagnosed CHD/heart attack/angina or prior CABG/PCI/stent	Weighted CHD prevalence 10.43% (9.91–10.96); women 8.39%, men 12.92%; age 40‐59 y 5.66%, ≥ 60 y 19.09%; urban 10.86%, rural 9.03%	Nationally representative for adults ≥ 40 y; self‐reported lifetime CHD.
**Registry‐ and hospital‐based surveillance evidence**						
Isfahan AMI/ACS surveillance network	Sarrafzadegan et al., 2009; Mohammadian‐Hafshejani et al., 2014; Mohammadian et al., 2015; Nouri et al., 2020; Bahrani et al., 2023; Paknahad et al., 2024 [32, 33, 34, 35, 36, 37]	Isfahan‐area hospitals; 2000–2017	Prospective/retrospective multicenter hospital surveillance and registry analyses	Hospitalized MI/IHD/ACS with 28‐day follow‐up in several reports	Admission rate 131.67 → 209.27/100 000 (2000–2004); 28‐day CFR women 23.1% versus men 13.2%; overall 28‐day CFR 11.2% → 7.9% (2000–2009); hospitalization‐derived IHD rates rose over 2001–2016; STEMI in‐hospital/28‐d mortality 6.5%/12.6%; 28‐day AMI mortality ~60% lower in 2014–2017 versus 2000–2002	Reports share an overlapping surveillance ecosystem. Hospital admission rates are not population incidence.
Iranian Myocardial Infarction Registry	Ahmadi et al., 2015 (EMHJ); Ahmadi et al., 2015 (J Res Med Sci); Ahmadi et al., 2015 (Int Cardiovasc Res J); Ahmadi et al., 2015 (J Mazandaran Univ Med Sci); Mozaffarian et al., 2021 [38, 39, 40, 41, 42]	Nationwide; registry years 2012–2014	National prospective/retrospective hospital registry analyses	First/new MI, ICD‐based; in‐hospital death and follow‐up survival	Age‐standardized MI incidence 73.3/100 000; provincial range 24.5–152.5. Among 20,750 MI admissions, 2511 in‐hospital deaths (12.1%); women 14.96% versus men 11.02% in the geographic mortality report. Highest standardized in‐hospital mortality incidence: Ardabil 31, North Khorasan 23.7, Yazd 20.2, West Azerbaijan 19.1, Khuzestan 18.4/100 000. First‐MI survival: 95% at 28 d, 90% at 6 mo, 88% at 1 y	Multiple reports from the same registry contribute distinct incidence, spatial, mortality, and survival outcomes; not independent populations.
Yazd hospital GIS study	Namayande et al., 2016 [43]	Three main Yazd hospitals; 2013; 440 admissions	Hospital‐admission GIS/spatial study	AMI hospital admissions	Reported overall rate 783/100 000 PY; men 531, women 458/100 000 PY	Overall rate is internally unusual relative to sex‐specific rates; interpret cautiously.
Tehran Heart Center STEMI database	Saadatagah et al., 2020 [44]	Tehran; Mar 2006–Mar 2017; *n* = 8295	Single tertiary‐center longitudinal STEMI database	STEMI	In‐hospital mortality declined 8.0% → 3.9%; length of stay 8.4 → 5.2 d	Long‐term hospital outcome trend; not population mortality.
Zanjan MI hospital registry	Soleimani & Bagheri, 2021 [45]	Nine hospitals; 2014–2018; *n* = 3820	Retrospective geospatial registry	Hospital‐registered MI	Overall reported age‐adjusted MI rate 343/100 000; annual rate 88 → 114/100 000 PY	Population denominator applied to hospital capture; not equivalent to cohort incidence.
IraPEN historical comparison	Ayat et al., 2022 [46]	Shahreza versus Najafabad; 2015 and 2019	Historical cohort from hospital information systems	Definite MI, ICD‐10 I21.0‐I21.9	Shahreza 1.85 → 2.43/1000 versus control 2.46 → 3.35/1000; difference‐in‐differences −0.31/1000; MI mortality difference ~−9/100 000	Policy evaluation; susceptible to secular/confounding effects.
Tehran pandemic AMI study	Pourasghari et al., 2023 [47]	Selected hospitals from three Tehran medical universities; 2019 versus 2020; *n* = 4560	Multicenter observational hospital study	STEMI/NSTEMI admissions	AMI admissions −12.65%; in‐hospital mortality 4.9% → 7.0% (OR 1.42, 95% CI 1.11–1.82)	Pandemic‐related hospital utilization/outcome evidence; not population incidence.
Birjand AMI hospital series	Kazemy & Sharifzadeh, 2006 [48]	1994–2003; Birjand hospitals	Retrospective hospital‐record study; 918 AMI hospitalizations	Definite hospitalized AMI	Annual AMI hospitalizations increased from 47 in 1994 to 201 in 2003	Hospital‐volume trend only; no population denominator and not interpretable as population incidence.
**Population‐based mortality evidence**						
Bushehr death registration	Omranikhoo et al., 2013 [49]	Bushehr Province; 2004–2011	Population mortality study using 29,916 registered deaths	IHD within avoidable‐mortality framework	IHD mortality declined approximately 8% per year in both rural and urban populations	Population death‐registration trend; coding/completeness limitations remain.
Central‐Iran mortality trend	Mehrabani‐Zeinabad et al., 2023 [50]	Isfahan intervention versus Arak control; 2000–2018	DRS + cemetery + census mortality trend with corrections	IHD cause‐specific mortality	Standardized IHD mortality in intervention area fell 44.62% during 2002–2011, then increased after 2011	Ecological comparison; avoid causal attribution to the intervention.
Fars death registration	Sharifi et al., 2024 [51]	Fars Province; 2004–2019	Population‐based death‐registration study	IHD deaths and YLL	46 969 IHD deaths; crude mortality 84.2/100 000 men and 66.5 women; YLL 287 625 men and 209 665 women	Direct subnational mortality/YLL evidence.
Mazandaran death registration	Khaleghi et al., 2026 [52]	Mazandaran Province; 2011–2023; 73 434 CVD deaths	Health authority + Civil Registration; joinpoint analysis	IHD ICD‐10 I20‐I25	IHD 31 135 deaths (42.39% of CVD deaths), the leading specific cardiovascular cause throughout	Recent empirical mortality evidence through 2023; cause‐of‐death misclassification remains possible.

*Note*: Population‐based incidence/prevalence, hospital admission, in‐hospital mortality, case fatality, post‐MI survival, and population mortality are distinct epidemiological constructs and were not considered interchangeable.

Abbreviations: ACS, acute coronary syndrome; AMI, acute myocardial infarction; CAD, coronary artery disease; CFR, case‐fatality rate; CHD, coronary heart disease; DRS, death registration system; ECG, electrocardiogram; ICD‐10, International Classification of Diseases, 10th Revision; IHD, ischemic heart disease; MI, myocardial infarction; NSTEMI, non‐ST‐segment elevation myocardial infarction; PCI, percutaneous coronary intervention; PY, person‐years; STEMI, ST‐segment elevation myocardial infarction; UAP, unstable angina pectoris.

In the Tehran Lipid and Glucose Study, crude CHD incidence was 11.9 per 1000 person‐years (95% CI: 10.6, 13.2) in men and 6.5 per 1000 person‐years (95% CI: 5.7, 7.3) in women; the corresponding Iran‐standardized rates were 10.5 and 6.1 per 1000 person‐years [34]. The MASHAD cohort similarly reported CHD incidence densities of 19.20 per 1000 person‐years (95% CI: 8.10, 30.30) in men and 11.60 per 1000 person‐years (95% CI: 7.30, 15.90) in women [54]. In the Yazd Healthy Heart Project, 225 incident CAD events occurred among 1,552 analyzed participants over a mean follow‐up of 9.9 years, giving a cumulative incidence of 14.5% (95% CI: 12.4, 16.6); sex‐specific incidence rates were 16.8 per 1000 person‐years (95% CI: 14.4, 19.2) in men and 12.0 per 1000 person‐years (95% CI: 9.5, 14.5) in women [56]. The principal national empirical estimate came from the Iranian Myocardial Infarction Registry, which recorded 20 750 new hospital‐registered MI cases in 2012 and estimated an age‐standardized MI rate of 73.3 per 100 000 (95% CI: 72.3, 74.3), with provincial rates ranging from 24.5 per 100 000 in Qom to 152.5 per 100 000 in North Khorasan [7]. Because the registry did not capture MI events that did not reach participating hospitals, this estimate was interpreted as registry‐derived incidence rather than complete population incidence (Supporting Information S1: Table 7).

#### 3.5.2 Prevalence

Empirical prevalence estimates varied substantially according to disease definition, ascertainment method, age range, and geographic setting and were therefore not pooled (Table 2; Supporting Information S1: Table 8). Among nationally representative studies, the SuRFNCD‐2011 survey estimated self‐reported CHD prevalence at 5.3% (95% CI: 4.6, 5.9) and chronic stable angina at 7.7% (95% CI: 4.6, 8.7) [19]. More recently, the Iran STEPS 2021 analysis of 16 552 adults aged ≥ 40 years reported a weighted CHD prevalence of 10.43% (95% CI: 9.91, 10.96), with estimates of 12.92% in men and 8.39% in women and a marked age gradient from 5.66% at ages 40–59 years to 19.09% at ≥ 60 years [53]. These national estimates are not directly comparable because the target populations and disease definitions differed.

Regional population studies also demonstrated substantial heterogeneity. In Tehran, age‐standardized CHD prevalence was 7.71% (95% CI: 7.18, 8.24) in the 2008–2011 TLGS analysis using adjudicated hospital records [12]. Self‐reported IHD prevalence was 10.2% (95% CI: 9.5, 10.9) in the Pars Cohort, while physician‐diagnosed self‐reported IHD prevalence was 5.7% in the Shahrekord PERSIAN Cohort and clinically confirmed IHD prevalence was 6.7% in the Hormozgan survey [20, 21, 29]. Other studies using broader ECG‐ or questionnaire‐based definitions reported higher estimates, including ECG‐defined nonfatal IHD prevalence of 12.7% in the Persian Gulf Healthy Heart Study and age‐adjusted composite CHD prevalence of 21.8% in an earlier TLGS analysis [10, 44]. These differences were interpreted primarily in the context of the underlying phenotype and ascertainment method rather than as direct geographic differences in disease prevalence (Supporting Information S1: Table 8).

### 3.6 Registry‐ and Hospital‐Based Surveillance

#### 3.6.1 Hospitalization and Acute Coronary Event Trends

Hospital‐based surveillance demonstrated substantial temporal and geographic variation in acute IHD and MI presentations, but these measures were interpreted as hospitalization or registry‐based event rates rather than population incidence (Table 2; Supporting Information S1: Table 9). Earlier hospital data from Birjand also showed an increase in the annual number of AMI admissions, from 47 in 1994 to 201 in 2003, although no population denominator was available [63]. In the Isfahan AMI surveillance network, the age‐adjusted hospital admission rate for MI increased from 131.67 per 100 000 in 2000 to 209.27 per 100 000 in 2004 [57]. A larger analysis of 102 254 IHD hospitalizations from the same regional surveillance system showed that hospitalization‐derived rates increased from approximately 27–37 per 10 000 in 2001–2002 to approximately 39–51 per 10 000 in 2015–2016, depending on the reference population and case definition, with estimated annual increases of 1.42%–3.36% [48].

Other regional registries showed similar variability. In Zanjan, 3820 hospital‐registered MI cases were identified between 2014 and 2018; the reported age‐adjusted rate was 343 per 100 000, while annual rates increased from 88 to 114 per 100 000 person‐years [61]. These estimates depended on hospital case capture and were therefore not considered equivalent to prospective population‐based incidence. During the COVID‐19 pandemic, a multicenter Tehran study reported a 12.65% reduction in AMI admissions in 2020 compared with 2019 [51].

#### 3.6.2 Case Fatality and Post‐MI Survival

Short‐term outcomes generally improved over time in longitudinal hospital‐surveillance datasets. In the Isfahan surveillance network, 28‐day MI case fatality was higher in women than in men (23.1% vs. 13.2%) during 2000–2004 [57]. In a subsequent analysis of first AMI, overall, 28‐day case fatality declined from 11.2% to 7.9% between 2000 and 2009; corresponding reductions were from 16.1% to 10.9% in women and from 9.2% to 6.7% in men [39]. A later Isfahan registry analysis reported approximately 60% lower 28‐day AMI mortality in 2014–2017 than in 2000–2002 [50].

The Iranian Myocardial Infarction Registry recorded 2511 in‐hospital deaths among 20 750 MI admissions, corresponding to a crude in‐hospital case‐fatality proportion of 12.1% [23]. A separate geographic analysis of the same registry reported higher crude case fatality among women than men (14.96% vs. 11.02%) and substantial provincial variation in standardized in‐hospital mortality rates [22]. Because these reports arose from the same national registry, they were treated as complementary analyses rather than independent populations.

Longitudinal hospital data also suggested improvements in acute outcomes. At Tehran Heart Center, in‐hospital mortality among patients with STEMI declined from 8.0% to 3.9% between 2006 and 2017 [52]. In the national first‐MI registry, 2479 of 21 181 patients died within 1 year (11.7%), with survival of 95% at 28 days, 90% at 6 months, and 88% at 1 year [43]. In contrast, during the COVID‐19 period, in‐hospital mortality among AMI admissions in Tehran increased from 4.9% in 2019 to 7.0% in 2020, corresponding to OR 1.42 (95% CI: 1.11, 1.82) [51]. These outcomes represent hospital case fatality or post‐MI survival and were not interpreted as population mortality (Supporting Information S1: Table 9).

### 3.7 Population‐Based Mortality and Disability Burden

#### 3.7.1 Empirical Mortality Trends

Population‐based mortality studies generally indicated substantial temporal and geographic variation in IHD mortality (Table 2; Supporting Information S1: Table 10). In Bushehr Province, registered IHD mortality declined by approximately 8% per year in both rural and urban populations between 2004 and 2011 [49]. In central Iran, standardized IHD mortality in the Isfahan intervention area declined by 44.62% during 2002–2011 but subsequently increased after 2011 [38]. Because this was an ecological comparison, the temporal change was not attributed solely to the intervention. In Fars Province, 46 969 IHD deaths were recorded between 2004 and 2019. Crude mortality rates were 84.2 per 100 000 in men and 66.5 per 100 000 in women, with corresponding YLL totals of 287 625 and 209 665 years, respectively [60]. More recent mortality surveillance from Mazandaran identified 31 135 IHD deaths between 2011 and 2023, representing 42.39% of all registered cardiovascular deaths and making IHD the leading specific cardiovascular cause throughout the study period [33].

#### 3.7.2 DALYs, YLLs, and YLDs

Burden‐of‐disease studies consistently showed that premature mortality accounted for most of the IHD disability burden. In GBD 2019, the age‐standardized DALY rate declined from 5437.6 to 2842.7 per 100 000 between 1990 and 2019; over the same period, the YLL rate declined from 5356.1 to 2761.7 per 100 000, whereas the YLD rate changed only slightly from 81.5 to 81.0 per 100 000 [8]. GBD 2021 similarly estimated a decline in the age‐standardized DALY rate from 5166.82 to 2731.30 per 100 000 between 1990 and 2021 [32].

Subnational burden studies also reported substantial IHD‐related health loss. In Isfahan, IHD accounted for 43517.71 DALYs in 2014, including 31891.79 YLLs and 11625.92 YLDs [59]. In West Azerbaijan, MI accounted for 53 804 DALYs, comprising 52 170 YLLs and 1634 YLDs [27], while in Kurdistan, IHD accounted for 71102.3 DALYs, representing 33.8% of total cardiovascular DALYs during 2011–2017 [41]. These estimates were interpreted within their original regional and methodological frameworks rather than directly compared with national GBD estimates (Table 1; Supporting Information S1: Table 10).

## 4 Discussion

This systematic review synthesized 52 reports spanning model‐based burden estimates, population‐based cohorts and surveys, national and regional registries, hospital surveillance systems, and death‐registration studies. Across successive GBD vintages, the most consistent national finding was a substantial decline in age‐standardized IHD mortality, accompanied by reductions in DALY rates, while absolute numbers of incident and prevalent cases increased markedly in the GBD 2019 analysis. Empirical evidence was more heterogeneous: prospective cohorts generally showed higher coronary event rates in men than women, whereas prevalence estimates varied widely according to age range, geographic setting, and whether disease was defined by self‐report, ECG criteria, clinical confirmation, or adjudicated outcomes.

The review also identified important demographic and geographic differences. GBD 2019 estimates showed higher age‐standardized incidence, prevalence, and mortality in men, while reports from the Isfahan surveillance network and the Iranian MI Registry indicated higher short‐term post‐MI case fatality among women. IHD burden increased sharply with age, and both modeled and empirical studies demonstrated substantial provincial variation. Hospital‐based surveillance suggested improving short‐term outcomes over time in several settings, including declining 28‐day and in‐hospital mortality, although these data cannot be interpreted as population mortality. Overall, the evidence indicates that Iran has experienced meaningful improvement in standardized IHD mortality, but the burden remains substantial and increasingly shaped by population aging, growth in absolute case numbers, geographic heterogeneity, and major differences in the completeness and comparability of available surveillance systems.

Despite substantial declines in age‐standardized IHD rates, absolute disease burden has increased. In GBD 2019, the age‐standardized incidence rate declined by 7.7% between 1990 and 2019, while the estimated number of incident cases increased by 171.8%, from 218 181 to 593 001 [8]. Similarly, GBD 2010 estimated a decline in age‐standardized mortality from 222 to 175 per 100 000 between 1990 and 2010, despite an increase in absolute deaths from 52 874 to 89 250 [37]. This divergence between standardized rates and absolute numbers is consistent with the effects of population growth and aging and has also been observed across countries undergoing epidemiological transition [1, 2, 3]. These findings emphasize that declining age‐standardized rates do not necessarily translate into a reduced absolute demand for IHD prevention and care [8, 37].

Recent national analyses indicate that the continuing burden of IHD in Iran occurs alongside a substantial burden of modifiable cardiovascular risk factors. GBD 2021‐based estimates showed that metabolic risk factors accounted for the largest share of cardiovascular DALYs and deaths in Iran, with high systolic blood pressure representing the leading individual contributor; high LDL cholesterol, high fasting plasma glucose, and high body mass index also remained important metabolic risks [64]. Although age‐standardized DALY and mortality rates attributable to cardiovascular risk factors declined between 1990 and 2021, the corresponding absolute numbers increased substantially [64]. Hypertension also remains incompletely controlled at the population level: national survey data showed that fewer than half of hypertensive adults were aware of their condition and only about one‐third were receiving pharmacological treatment in 2011 [65]. Overall, substantial opportunities remain for improving primary prevention and cardiovascular risk‐factor detection and management in Iran [64, 65]. Iran has already established national frameworks for this purpose, including the national NCD action plan and Ira‐PEN cardiovascular risk assessment, but sustained implementation and coverage remain important [66, 67, 68].

Age‐standardized IHD prevalence has changed relatively little compared with mortality. In GBD 2019, the age‐standardized prevalence rate declined only slightly, from 6250.6 per 100 000 in 1990 to 6198.5 per 100 000 in 2019 [8]. Empirical studies also demonstrated substantial prevalence, although estimates varied considerably according to age range, disease definition, and ascertainment method. Urban‐rural patterns were not consistent across studies: prevalence was somewhat higher in urban populations in SuRFNCD‐2011, the Shahrekord PERSIAN Cohort, and the Hormozgan survey, whereas the Tabari Cohort reported higher prevalence in rural participants [19, 21, 29, 30]. IHD burden also increased markedly with age. In GBD 2019, adults aged ≥ 70 years had an estimated IHD prevalence of 52886.4 per 100 000 and a mortality rate of 1932.9 per 100 000 in 2019 [42]. National STEPS 2021 data likewise showed a strong age gradient in CHD prevalence, from 5.66% among adults aged 40–59 years to 19.09% among those aged ≥ 60 years [53]. Therefore, population aging is likely to sustain substantial demand for IHD prevention, chronic disease management, and acute cardiovascular care.

Sex‐related differences were observed across several sources. In GBD 2019, men had higher age‐standardized incidence, prevalence, and mortality rates than women [8]. In contrast, multiple reports arising from two registry/surveillance source families, the Isfahan surveillance network and the Iranian MI Registry, reported higher short‐term post‐MI case fatality among women. In the Isfahan surveillance network, 28‐day case fatality was 23.1% in women versus 13.2% in men [57], and a later analysis from the same surveillance system showed persistently higher 28‐day case fatality among women during 2000–2009 [39]. A geographic analysis from the Iranian MI Registry reported a higher crude in‐hospital case‐fatality proportion among women than men (14.96% vs. 11.02%) [22]. These findings are consistent with broader literature describing sex differences in IHD presentation and outcomes [69]. However, because the registry findings were derived from a limited number of underlying surveillance sources, they should not be interpreted as independent national confirmation of a uniform sex difference in post‐MI prognosis.

Substantial geographic variation was evident across both modeled and empirical evidence. Within GBD 2019, North Khorasan had the highest provincial age‐standardized IHD incidence rate, whereas Golestan had the highest mortality rate [11]. In the GBD 2023 mortality analysis, age‐standardized IHD mortality ranged from 88.1 per 100 000 in Tehran to 262.8 per 100 000 in Qom [58], while the national MI registry reported registry‐derived MI rates ranging from 24.5 per 100 000 in Qom to 152.5 per 100 000 in North Khorasan [7]. These findings demonstrate substantial subnational heterogeneity, although provincial rankings differed according to outcome, data source, and model vintage. Evidence regarding urban‐rural differences was less consistent: several studies reported somewhat higher prevalence in urban populations, whereas the Tabari Cohort reported higher prevalence in rural participants [19, 21, 29, 30]. The available evidence therefore does not support a uniform urban‐rural gradient in IHD burden.

Iran has implemented several national initiatives in acute IHD care, including a 24/7 primary PCI program and nationwide MI registry infrastructure [7, 70]. Hospital‐based studies also documented declining short‐term mortality in several settings; for example, in‐hospital mortality among patients with STEMI at Tehran Heart Center decreased from 8.0% to 3.9% between 2006 and 2017 [52], while longitudinal surveillance in Isfahan showed substantial reductions in 28‐day mortality after AMI [39, 50]. These findings indicate favorable temporal trends in hospital‐based acute IHD outcomes, although they should not be interpreted as direct evidence that healthcare‐system changes caused the decline in population‐level IHD mortality. Moreover, GBD 2019 showed much smaller reductions in age‐standardized incidence and prevalence than in mortality [8], showing that declines in fatal burden were not paralleled by comparable reductions in nonfatal IHD burden.

### 4.1 Strengths and Limitations

Important strengths of this review include the systematic identification of evidence, explicit separation of modeled and empirical estimates, design‐specific methodological appraisal, mapping of overlapping data sources, and distinction between population incidence and mortality, hospitalization, and case‐fatality measures. This review has several limitations. Much of the national evidence derives from modeled estimates, while empirical studies varied in geographic coverage, disease definitions, denominators, and ascertainment methods. Several prevalence studies relied on self‐report or ECG‐/questionnaire‐based definitions, and registry or hospital‐surveillance studies captured only patients reaching participating facilities, limiting population‐level inference. Hospital‐ and registry‐derived measures may also differ from population‐based incidence and mortality because of variation in healthcare access, referral and admission practices, hospital case capture, completeness and coding of death registration, denominator selection, and follow‐up duration; these measures were therefore not considered directly interchangeable. Geographic coverage was also uneven, particularly for some provinces and rural populations. Heterogeneity across studies precluded quantitative pooling, and incidence, prevalence, hospitalization, case fatality, and population mortality could not be treated as interchangeable outcomes. In addition, successive GBD releases used different source data and modeling approaches and were therefore not interpreted as a single directly comparable time series. Overlap among several cohorts, registries, and GBD‐derived reports also required explicit source mapping. This review was restricted to PubMed and Scopus. Consequently, some locally indexed or gray literature may have been missed. Therefore, more comprehensive national cardiovascular surveillance with standardized case definitions, improved linkage of hospital, primary‐care, and mortality data, and broader representation of underserved geographic areas are required.

## 5 Conclusion

In summary, within GBD 2019, age‐standardized IHD mortality declined by 42.7%, from 285.7 to 163.6 per 100 000 between 1990 and 2019, while age‐standardized incidence declined by 7.7% and the estimated number of incident cases increased by 171.8%, from 218 181 to 593 001. Empirical studies complemented these model‐based findings: prospective cohorts generally reported higher coronary event rates in men, whereas 28‐day MI case fatality in the Isfahan surveillance network was higher in women than men (23.1% vs. 13.2%). Hospital outcomes also improved in several settings, including a decline in in‐hospital STEMI mortality from 8.0% to 3.9% at Tehran Heart Center. Despite these improvements, the absolute burden remains large and substantial differences remain by age, sex, and geography. Continued progress will require stronger primary prevention and cardiometabolic risk‐factor control, sustained improvements in acute and secondary care, and more comprehensive national surveillance using standardized case definitions and geographically representative data.

## Author Contributions

**Mehrdad Rabiee Rad:** conceptualization, methodology, writing – original draft, writing – review and editing; **Bahar Darouei:** methodology, data curation, visualization, writing – original draft; **Ghazal Ghasempour Dabaghi:** data curation, writing – original draft; **Reza Amani‐Beni:** data curation, visualization, writing – review and editing; **Fahimeh Bagheri, Shahla Shahidi, Noushin Mohammadifard, Fatemeh Sabeti, Sana Azizian,** and **Ali Pourmoghaddas:** data curation, writing – review and editing; **Shahin Shirani:** methodology, supervision, validation, writing – review and editing; **Mohammadreza Sabri:** supervision, validation, writing – review and editing; **Nizal Sarrafzadegan:** conceptualization, methodology, project administration, resources, supervision, writing – review and editing. All authors have reviewed the manuscript.

## Funding

The authors have nothing to report.

## Ethics Statement

This study involved a secondary analysis of publicly available data and published literature, and did not require ethical approval.

## Consent

The authors have nothing to report.

## Conflicts of Interest

The authors declare no conflicts of interest.

## Supporting information


Supporting File

## Data Availability

No new primary data were generated. All data extracted for this review were obtained from the cited reports. The study‐level extraction sheet, evidence‐family overlap map, appraisal judgments, search strategies, and selection records are available in Supplementary Material.
