# Supplementary material for: National and Subnational Trends in Ischemic Heart Disease Burden in Iran, 1990–2023: A Systematic Review
Source: Clin Cardiol. 2026 Oct 7;49(10):e70487. doi: 10.1002/clc.70487 (PMC13644550; doi:10.1002/clc.70487)
Supplement: Supplementary file 1 [file CLC-49-e70487-s001.docx]

**SUPPLEMENTARY TABLES**

Table of Contents

[Table S1. Prespecified eligibility criteria for study inclusion.	2](#_Toc237776103)

[Table S2. Electronic database and supplementary search strategies used to identify studies on the burden of ischemic heart disease in Iran.	3](#_Toc237776104)

[Table S3. Full-text reports excluded after detailed assessment.	4](#_Toc237776105)

[Table S4. Overlapping study populations, registries, surveillance systems, and GBD model vintages.	7](#_Toc237776106)

[Table S5. Disease definitions and outcome ascertainment across the principal data sources.	8](#_Toc237776107)

[Table S6. Design-specific methodological appraisal of included reports.	10](#_Toc237776108)

[Table S7. Detailed incidence estimates for ischemic heart disease and myocardial infarction in Iran.	16](#_Toc237776109)

[Table S8. Detailed prevalence estimates for ischemic heart disease, coronary heart disease, coronary artery disease, and myocardial infarction in Iran.	17](#_Toc237776110)

[Table S9. Hospitalization and acute coronary surveillance estimates.	19](#_Toc237776111)

[Table S10. Detailed mortality, case-fatality, and burden-of-disease estimates.	21](#_Toc237776112)

[Table S10. Detailed mortality, case-fatality, and burden-of-disease estimates (continued).	23](#_Toc237776113)

# Table S1. Prespecified eligibility criteria for study inclusion.

| **Domain** | **Inclusion criteria** | **Exclusion criteria** |
| --- | --- | --- |
| Population and setting | Studies reporting data from populations residing in Iran, including national, provincial, district, community, registry, and hospital-based populations. | Studies outside Iran; Iranian migrants outside Iran; multinational studies without separable Iran-specific results. |
| Target condition | IHD, CHD, CAD, MI, or clearly defined acute coronary manifestations when they contributed an eligible burden measure. | Broad composite CVD without separable IHD/CHD/CAD/MI; non-IHD cardiac or vascular conditions. |
| Primary burden outcomes | Population incidence/cumulative incidence/incidence density, prevalence, population mortality, age-standardized rates, absolute cases/deaths, and temporal/geographic trends. | No extractable epidemiological burden measure. |
| Secondary burden outcomes | Hospital admission/hospitalization trends; in-hospital mortality; short-term case fatality; longer-term post-MI survival when derived from an eligible registry/surveillance population. | Clinical studies reporting outcomes solely for prognosis, treatment efficacy, procedure performance, or predictors without a surveillance/burden objective. |
| Burden-of-disease measures | DALYs, YLLs, YLDs, and other directly relevant IHD burden measures from GBD or national/subnational burden studies. | Risk-factor-attributable burden when overall IHD burden was not directly estimated. |
| Study designs | Population-based cross-sectional studies; prospective/retrospective population cohorts; national/regional disease registries; death-registration studies; multicenter or population-catchment hospital surveillance; ecological/time-trend studies; GBD or other population-level modeling studies.  Hospital-based studies without a population denominator were eligible only when they evaluated an explicit temporal or geographic surveillance objective, including longitudinal admission or case-fatality trends, registry-based geographic comparisons, or multicenter comparisons of major health-system disruptions. | Case reports/series without denominator, qualitative studies, trials, treatment/intervention studies, diagnostic accuracy studies, purely laboratory/genetic/biomarker studies. |
| Risk-factor/etiological studies | Eligible only if the underlying study also provided a directly usable population-based burden estimate relevant independently of the exposure analysis. | Exposure-association studies without a unique burden estimate. |
| Clinical/procedural populations | Registry/surveillance studies retained when they quantified hospitalization patterns, case fatality, temporal or geographic burden in a defined catchment. | Convenience samples undergoing angiography, PCI, CABG, rehabilitation, or other procedures without a meaningful surveillance/population denominator. |
| Disease ascertainment | Clinical criteria, registry definitions, ICD codes, ECG criteria, physician diagnosis, validated survey definitions, or self-reported physician-diagnosed disease with clear methods. | Unclear/non-reproducible disease definitions. |
| Geographic level | National and subnational evidence; regional/city-specific studies with defined source population/catchment. | Undefined catchment or highly selected clinical population without meaningful inference. |
| Time period | Data relevant to 1990–2023. Articles published after 2023 were eligible if they analyzed data from this period. | No data relevant to the prespecified burden period. |
| Publication date/language | No publication-date or language restriction at the search stage; English- or Persian-language reports considered when sufficient information could be obtained. | Reports in which eligibility/outcome information could not be reliably determined. |
| Publication type | Full original research articles, full-text preprints and formal population-level burden/modeling reports. | Reviews/meta-analyses, editorials, letters without eligible original data, conference abstracts without sufficient data, protocols, commentaries, corrections. |
| Overlapping populations | Multiple reports from the same source could be retained when they contributed distinct eligible outcomes, time periods, subgroups, or geography. | Redundant reports with no unique eligible information were excluded; overlapping reports were not treated as independent populations. |

IHD, CHD, CAD, MI, ACS, STEMI, NSTEMI, or unstable angina when they contributed an eligible population-level, registry, surveillance, hospitalization, case-fatality, survival, or burden measure.

# Table S2. Electronic database and supplementary search strategies used to identify studies on the burden of ischemic heart disease in Iran.

| **Source** | **Search date** | **Search strategy** | **Coverage and limits** | **Records retrieved/screened** |
| --- | --- | --- | --- | --- |
| PubMed | 15 August 2026 | ("Myocardial Ischemia"[Mesh] OR "Coronary Artery Disease"[Mesh] OR "Myocardial Infarction"[Mesh] OR "Acute Coronary Syndrome"[Mesh] OR "Angina, Unstable"[Mesh] OR "ischemic heart disease"[tiab] OR "ischaemic heart disease"[tiab] OR "coronary heart disease"[tiab] OR "coronary artery disease"[tiab] OR "myocardial infarction"[tiab] OR "acute myocardial infarction"[tiab] OR "acute coronary syndrome"[tiab] OR ACS[tiab] OR STEMI[tiab] OR NSTEMI[tiab] OR "ST-elevation myocardial infarction"[tiab] OR "ST elevation myocardial infarction"[tiab] OR "non-ST-elevation myocardial infarction"[tiab] OR "non-ST elevation myocardial infarction"[tiab] OR "unstable angina"[tiab]) AND ("Iran"[Mesh] OR Iran[tiab] OR Iranian[tiab]) AND ("Incidence"[Mesh] OR "Prevalence"[Mesh] OR "Mortality"[Mesh] OR incidence[tiab] OR prevalence[tiab] OR mortality[tiab] OR epidemiolog*[tiab] OR burden[tiab] OR trend*[tiab] OR "case fatality"[tiab] OR "case-fatality"[tiab]) | Database inception to search date. No language or publication-type restrictions at the search stage. | 931 |
| Scopus | 15 August 2026 | TITLE-ABS-KEY(("ischemic heart disease" OR "ischaemic heart disease" OR "coronary heart disease" OR "coronary artery disease" OR "myocardial infarction" OR "acute myocardial infarction" OR "acute coronary syndrome" OR ACS OR STEMI OR NSTEMI OR "ST-elevation myocardial infarction" OR "ST elevation myocardial infarction" OR "non-ST-elevation myocardial infarction" OR "non-ST elevation myocardial infarction" OR "unstable angina") AND (Iran OR Iranian) AND ("Incidence" OR "Prevalence" OR "Mortality" OR incidence OR prevalence OR mortality OR epidemiolog* OR burden OR trend* OR "case fatality" OR "case-fatality") | Database inception to search date. No language or document-type restrictions at the search stage. | 1613 |
| Google Scholar | 15 August 2026 | ("ischemic heart disease" OR "coronary heart disease" OR "coronary artery disease" OR "myocardial infarction" OR "acute coronary syndrome" OR STEMI OR NSTEMI OR "unstable angina") Iran (incidence OR prevalence OR mortality OR hospitalization OR admission OR registry OR surveillance OR "case fatality" OR survival OR burden OR trend) | Supplementary search source. The first 200 results ranked by relevance were screened to identify potentially missed eligible studies and citation-linked literature. | 200 screened |

# Table S3. Full-text reports excluded after detailed assessment.

| **Report** | **Year** | **Final full-text decision/reason** | **PRISMA exclusion category** |
| --- | --- | --- | --- |
| Beyranvand et al. | 2007 | Exclude: small single-hospital AMI prognosis/readmission study without a population or surveillance burden denominator. | Risk-factor/etiologic/prognostic/selected clinical study |
| Ghadimi et al. | 2006 | Exclude: small single-center AMI cohort focused on complications and 1-year outcome. | Risk-factor/etiologic/prognostic/selected clinical study |
| Nabipour et al. (second overlapping report) | 2008 | Exclude as overlapping/redundant: same Persian Gulf Healthy Heart Study population and same ECG-IHD prevalence as the retained 2006 report. | Overlapping/redundant |
| Talaei et al. (metabolic syndrome analysis) | 2012 | Exclude: etiological/risk-factor analysis from ICS; direct burden reports from the same cohort are retained. | Risk-factor/etiologic/prognostic/selected clinical study |
| Sarrafzadegan et al. (rural/urban composite CVD) | 2012 | Exclude: composite CVD incidence without a separable IHD incidence outcome. | Ineligible/composite/insufficiently interpretable |
| Hafshejani et al. | 2012 | Exclude: sex-specific predictors of 28-day AMI survival; overlapping prognostic analysis without unique burden trend. | Risk-factor/etiologic/prognostic/selected clinical study |
| Mohammadian-Hafshejani et al. | 2012 | Exclude: predictor analysis of short-term AMI survival from Isfahan registry; no unique burden estimate. | Risk-factor/etiologic/prognostic/selected clinical study |
| Bozorgmanesh et al. | 2013 | Exclude: restricted diabetic population and hypertension-attributable composite CVD risk rather than general IHD burden. | Risk-factor/etiologic/prognostic/selected clinical study |
| Ghaffari et al. | 2013 | Exclude: selected AMI clinical cohort focused on treatment/complications/outcomes rather than burden surveillance. | Risk-factor/etiologic/prognostic/selected clinical study |
| Ehteshami-Afshar et al. | 2014 | Exclude: smoking-focused TLGS etiological analysis; direct TLGS burden reports retained. | Risk-factor/etiologic/prognostic/selected clinical study |
| Shahraz et al. | 2014 | Exclude: broad GBD regional comparison without a unique focused Iran-specific IHD estimate beyond retained reports. | Overlapping/redundant |
| Forouzanfar et al. | 2014 | Exclude: broad GBD 2010 causes-of-death/morbidity report; no uniquely necessary IHD estimate beyond focused retained report. | Overlapping/redundant |
| Ahmadi et al. (education/risk-factor mortality analysis) | 2014/2015 | Exclude: secondary national MI-registry risk-factor analysis with no unique burden estimate. | Risk-factor/etiologic/prognostic/selected clinical study |
| Nejatinamini et al. | 2015 | Exclude: IHD burden attributable specifically to low omega-3 intake rather than overall IHD burden. | Risk-factor/etiologic/prognostic/selected clinical study |
| Some Facts about Case Fatality of Acute Myocardial Infarction in Iran | 2015 | Exclude: letter/commentary, not an original eligible burden study. | Letter/non-original |
| Ahmadi et al. (Kardiologia Polska first-registry report) | 2015 | Exclude as redundant registry report: the same 20,750-patient registry outcomes are captured more directly by retained incidence, spatial, CFR, and geographic-mortality reports. | Overlapping/redundant |
| Kassaian et al. (IPACE2) | 2015 | Exclude: selected tertiary-center patients discharged alive; principally post-ACS management/prognosis rather than burden surveillance. | Risk-factor/etiologic/prognostic/selected clinical study |
| Ahmadi et al. (age at first MI) | 2016 | Exclude: age-at-first-MI analysis; age at event is not a prespecified burden outcome. | Risk-factor/etiologic/prognostic/selected clinical study |
| Hadei et al. | 2017 | Exclude: PM2.5-attributable IHD deaths rather than overall IHD burden. | Risk-factor/etiologic/prognostic/selected clinical study |
| Aghamohamadi et al. | 2017 | Exclude: broad national leading-causes-of-death paper without a sufficiently focused extractable IHD burden analysis. | Ineligible/composite/insufficiently interpretable |
| Naghavi et al. (health-system/GBD performance) | 2022 | Exclude: broad health-system/GBD performance report; focused IHD GBD reports available. | Overlapping/redundant |
| Mansourian et al. | 2022 | Exclude: comorbidity burden modeling, not overall IHD burden. | Ineligible/composite/insufficiently interpretable |
| Roudkoli et al. | 2022 | Exclude: selected MINOCA clinical population; clinical/risk-factor characterization rather than population burden. | Risk-factor/etiologic/prognostic/selected clinical study |
| Abbasi et al. (rural/urban ACS mortality) | 2022 | Exclude: hospitalized ACS comparison without a population denominator or unique temporal burden trend. | Risk-factor/etiologic/prognostic/selected clinical study |
| Kohi et al. (IMPACT model) | 2023 | Exclude from core evidence: explanatory counterfactual decomposition of treatment/risk-factor contributions rather than a direct IHD burden estimate; may be cited in Discussion. | Risk-factor/etiologic/prognostic/selected clinical study |
| Darabi et al. | 2023 | Exclude: Kermanshah STEMI age/sex prognosis analysis; selected clinical registry and overlapping outcomes. | Risk-factor/etiologic/prognostic/selected clinical study |
| Sarraf-Zadegan et al. | 1999 | Exclude as redundant registry report | Overlapping/redundant |
| Sarraf-Zadegan et al. | 1999 | Exclude as redundant registry report | Overlapping/redundant |
| Nalini et al. | 2018 | Exclude: IHD proportion among premature deaths without a directly usable IHD population mortality rate. | Ineligible/composite/insufficiently interpretable |
| Siabani et al. | 2020 | Exclude: sex-specific STEMI mortality/predictor analysis from selected registry; no unique burden trend. | Risk-factor/etiologic/prognostic/selected clinical study |
| Alemzade-Ansari et al. | 2020 | Exclude: single-center ACS registry focused on procedural/acute clinical outcomes without a population or longitudinal surveillance burden objective. | Risk-factor/etiologic/prognostic/selected clinical study |
| Kabootari et al. | 2018 | Exclude: weight-history exposure study with incident CHD/stroke as etiological outcomes; direct TLGS burden reports retained. | Risk-factor/etiologic/prognostic/selected clinical study |
| Hosseini et al. | 2009 | Exclude: young STEMI clinical characteristics/management/outcome study; selected clinical population. | Risk-factor/etiologic/prognostic/selected clinical study |
| Abbasi et al. (selected clinical CAD) | 2012 | Exclude: selected clinical CAD population examining sex/risk factors; not a population burden study. | Risk-factor/etiologic/prognostic/selected clinical study |
| Zeinali-Nezhad et al. | 2024 | Exclude: prevalence/incidence of CAD risk factors rather than CAD/IHD disease burden. | Risk-factor/etiologic/prognostic/selected clinical study |
| Sharaf et al. | 2024 | Exclude: STEMI/NSTEMI percentages derived from MI-registry cases plus selected healthy controls; not population prevalence. | Ineligible/composite/insufficiently interpretable |
| Soleimani et al. (regional total-CVD GBD) | 2024 | Exclude: broad total-CVD burden report without a sufficiently central/separable Iran-specific IHD estimate for the core evidence set. | Ineligible/composite/insufficiently interpretable |
| Sarebanhassanabadi et al. (uric-acid analysis) | 2025 | Exclude as redundant/etiological: same 1,552 Yazd cohort participants and 225 CAD events as retained 2024 burden report. | Overlapping/redundant |
| Hosseininezhad et al. | 2025 | Exclude from core evidence: single referral center and two short pandemic windows; insufficiently robust as a burden-surveillance source. | Ineligible/composite/insufficiently interpretable |
| Safiri et al. (global GBD 2019 IHD analysis) | 2022 | Exclude as redundant model derivative: Iran estimates are already represented by the focused Koolaji GBD 2019 report. | Overlapping/redundant |
| Sadeghi et al. (EMR GBD 2019 analysis) | 2023 | Exclude as redundant regional model derivative: Iran estimates use the same GBD 2019 model vintage and add no necessary unique core estimate. | Overlapping/redundant |
| Fakhrzadeh et al. (earlier Qazvin report) | 2005 | Exclude as overlapping/redundant with the retained fuller Qazvin population report. | Overlapping/redundant |

# Table S4. Overlapping study populations, registries, surveillance systems, and GBD model vintages.

| **Underlying source/family** | **Included reports** | **Years/coverage** | **Outcomes contributed** | **Overlap assessment and final handling** |
| --- | --- | --- | --- | --- |
| TLGS | Ghanbarian 2004; Hadaegh 2009; Khalili 2014; Moazzeni 2020 | Tehran baseline waves and follow-up through ~2010/2011 | ECG MI, CHD prevalence, CHD incidence | Shared cohort framework; retained because reports use different waves/definitions/outcomes. Synthesized as one source family in Main Table 2. |
| IHHP / ICS | Sadeghi 2006; Sarrafzadegan 2011; Talaei 2013 | Central Iran baseline + prospective follow-up | CAD prevalence, IHD/AMI/UAP/SCD incidence | Same research program with distinct baseline/follow-up outcomes. Synthesized as one source family. |
| Qazvin population study | Fakhrzadeh 2008 | Early/mid-2000s | Possible MI, ischemic ECG, angina prevalence | Earlier 2005 report was excluded as overlapping/redundant; 2008 report retained as the core Qazvin source. |
| Yazd Healthy Heart Project | Sarebanhassanabadi 2024 | 2005–2006 baseline; ~9.9 y follow-up | Incident CAD | 2025 uric-acid paper used the same 1,552 participants/225 CAD events and was excluded as redundant etiological analysis. |
| Isfahan AMI/ACS surveillance network | Sarrafzadegan 2009; Mohammadian-Hafshejani 2014; Mohammadian 2015; Nouri 2020; Bahrani 2023; Paknahad 2024 | 2000–2017 | Admissions, hospitalization trends, 28-d CFR, ACS mortality | Substantial temporal/population overlap. Retained only where reports add distinct time windows/outcomes; treated as one source family in Main Table 2. |
| Iranian MI Registry | Ahmadi 2015 EMHJ; Ahmadi 2015 J Res Med Sci; Ahmadi 2015 Int Cardiovasc Res J; Ahmadi 2015 J Mazandaran; Mozaffarian 2021 | 2012–2014 | National MI incidence, spatial incidence, in-hospital CFR, geographic in-hospital mortality, post-MI survival | Same registry program. Reports retained for unique outcomes, but not treated as independent populations. |
| GBD 2019 model vintage | Koolaji 2023; Mousavi 2023; Karamitanha 2024; Aminorroaya 2024 | 1990–2019 | National all-age burden, ≥70-y burden, provincial incidence/mortality, NAME comparisons, and decomposition of changes in incident cases. | Same GBD 2019 estimates. Synthesized as one model family in Main Table 1. |
| Other GBD vintages | Maracy 2015 (GBD 2010); Heidari-Foroozan 2025 (GBD 2021); Sepanlou 2026 (GBD 2023) | 1990–2010/2021/2023 | Mortality/DALY and selected nonfatal outcomes | Separate model vintages; not considered independent observed epidemiological series and not directly compared across releases without caveat. |
| Central-Iran mortality evidence | Kiani 2019; Mehrabani-Zeinabad 2023 | Approx. 2000–2018 | IHD mortality modeling vs corrected observed mortality trend | Partly overlapping geographic/time context but different data-processing/modeling approaches; retained as methodologically distinct evidence, not mutual validation. |

Overlap was assessed at the underlying cohort, registry, surveillance-system, survey, or GBD-model-vintage level. Multiple reports were retained only when they contributed distinct eligible information and were not treated as independent populations.

# Table S5. Disease definitions and outcome ascertainment across the principal data sources.

| **Source/dataset** | **Included report(s)** | **Reported term** | **Operational definition/criteria** | **Ascertainment** | **Key caveat** |
| --- | --- | --- | --- | --- | --- |
| GBD 2010/2019/2021/2023 | Maracy 2015; Koolaji 2023; Mousavi 2023; Karamitanha 2024; Heidari-Foroozan 2025; Sepanlou 2026; Aminorroaya 2024 | IHD | GBD cause hierarchy; mapped ICD inputs, generally I20–I25 and related source codes | Vital registration, surveys, hospital/claims and other inputs with statistical modeling | Model vintages differ; release-to-release values are not directly observed surveillance trends. |
| Iran NBD 2003 | Naghavi 2009 | IHD | National burden cause category | National mortality/morbidity inputs + burden modeling | Older methodology; not directly comparable with GBD. |
| TLGS | Ghanbarian 2004; Hadaegh 2009; Khalili 2014; Moazzeni 2020 | MI/CHD | Minnesota ECG, Rose angina/self-report, later adjudicated CHD | Population survey and prospective follow-up | Phenotype and ascertainment changed across waves. |
| IHHP/ICS | Sadeghi 2006; Sarrafzadegan 2011; Talaei 2013 | CAD/IHD/AMI/UAP/SCD | Rose + Minnesota ECG at baseline; adjudicated clinical outcomes in follow-up | Population survey + prospective cohort | Do not equate broad baseline CAD with adjudicated incident IHD. |
| Persian Gulf Healthy Heart Study | Nabipour 2006 | Nonfatal IHD | Minnesota ECG ischemic changes/MI | Population ECG survey | ECG-defined disease may include silent/subclinical disease. |
| Qazvin population study | Fakhrzadeh 2008 | Possible MI / ischemic ECG / angina | Survey + ECG criteria | Population cross-sectional survey | Outcomes are separate constructs. |
| SuRFNCD-2011 | Abbasi 2018 | CHD / stable angina | Self-reported CHD; angina questionnaire | National survey | Self-report; CHD and angina not interchangeable. |
| Borujerd study | Maleki 2019 | Probable/definite CAD | Rose + Minnesota ECG + documented CAD history | Population survey | Multiple diagnostic components; abstract/table inconsistency. |
| Tabari/Pars/Shahrekord PERSIAN | Ghaemian 2020; Adib 2020; Ahmadi 2021 | CHD/IHD/MI | Self-reported physician/history-based disease | Population cohort baselines | Potential recall/diagnostic-access bias. |
| MASHAD/Yazd prospective cohorts | Sadabadi 2022; Sarebanhassanabadi 2024 | Incident CHD/CAD | Clinical/adjudicated coronary events | Prospective follow-up | Regional cohorts; Yazd has attrition. |
| Hormozgan survey | Farshidi 2025 | IHD | Self-report followed by record review/cardiologist confirmation | Population survey | Subnational only. |
| Iran STEPS 2021 | Sabbagh Alvani 2026 | CHD | Self-reported physician-diagnosed heart attack/angina or CABG/PCI/stent | National STEPS survey | Age ≥40 only; lifetime self-report. |
| Isfahan AMI/ACS surveillance | Sarrafzadegan 2009; Mohammadian-Hafshejani 2014; Mohammadian 2015; Nouri 2020; Bahrani 2023; Paknahad 2024 | MI/IHD/ACS | WHO-MONICA/ICD/registry criteria; 28-day follow-up in several reports | Hospital surveillance network | Hospital admission and CFR are not population incidence/mortality. |
| Iranian MI Registry | Ahmadi 2015 EMHJ; J Res Med Sci; Int Cardiovasc Res J; J Mazandaran; Mozaffarian 2021 | First/new MI and in-hospital death | WHO/WHF + ICD-10 I21–I22 and registry criteria | National hospital registry | Misses pre-hospital events/deaths; multiple publications overlap. |
| Other hospital surveillance | Namayande 2016; Saadatagah 2020; Soleimani & Bagheri 2021; Ayat 2022; Pourasghari 2023; Kazemy & Sharifzadeh 2006 | AMI/STEMI/MI | Hospital clinical/ICD diagnoses | Hospital registries/information systems | Selection/catchment and denominator limitations. |
| Death-registration studies | Omranikhoo 2013; Mehrabani-Zeinabad 2023; Sharifi 2024; Khaleghi 2026 | IHD mortality | Cause-of-death coding, generally ICD-based | DRS/Civil Registration/health authority data | Completeness and cause-of-death misclassification vary by place/time. |
| Other burden/mortality models | Shams-Beyranvand 2017; Ebrahimi 2017; Kiani 2019; Moradi 2021; Naderimagham 2022 | IHD/MI mortality and DALY/YLL/YLD | Study-specific burden/model definitions | Regional/national mortality and morbidity inputs | Model assumptions and input-data completeness differ. |

# Table S6. Design-specific methodological appraisal of included reports.

| **Study/report** | **Appraisal framework** | **Sampling / data-source coverage** | **Outcome / input ascertainment** | **Completeness / follow-up / transparency** | **Analysis / denominator / uncertainty** | **Overall appraisal and key limitation** |
| --- | --- | --- | --- | --- | --- | --- |
| **Population-based prevalence and incidence studies** | | | | | | |
| **Ghanbarian et al., 2004** | JBI Prevalence Studies | Some concern: population-based Tehran sample, but antihypertensive-treated adults were excluded. | Some concern: Minnesota-coded resting ECG identifies ECG-defined MI rather than all clinically recognized MI. | Adequate for cross-sectional analysis; response/coverage limitations should be considered. | Adequate for reported prevalence; phenotype-specific denominator used. | Some methodological concerns; useful for ECG-defined MI prevalence, not clinical MI prevalence. |
| **Sadeghi et al., 2006** | JBI Prevalence Studies | Adequate multistage population sampling in Isfahan, Najafabad, and Arak; not nationally representative. | Some concern: broad Rose questionnaire plus Minnesota ECG definition increases heterogeneity of CAD classification. | Adequate for cross-sectional survey. | Adequate for prevalence estimation within sampled population. | Some methodological concerns, mainly case-definition breadth and regional generalizability. |
| **Nabipour et al., 2006** | JBI Prevalence Studies | Adequate cluster-random population sample from three Persian Gulf ports; regional coverage only. | Some concern: Minnesota ECG-defined IHD differs from clinically adjudicated IHD. | Adequate for cross-sectional survey. | Adequate for reported ECG-IHD prevalence. | Some methodological concerns; robust regional sampling but phenotype is ECG-defined. |
| **Fakhrzadeh et al., 2008** | JBI Prevalence Studies | Some concern: population-based Qazvin sample with moderate sample size. | Some concern: several distinct constructs (possible MI, ischemic ECG changes, angina) measured separately. | Adequate for cross-sectional survey. | Adequate when outcomes are kept separate; inappropriate to combine as one CHD estimate. | Some methodological concerns; estimates are usable when individual outcome definitions are preserved. |
| **Hadaegh et al., 2009** | JBI Prevalence Studies | Adequate population-based TLGS sampling; Tehran only. | Some concern: composite CHD definition combines Rose angina, self-report, and Minnesota ECG criteria. | Adequate for cross-sectional analysis. | Adequate for the prespecified composite outcome. | Some methodological concerns due to broad composite case definition. |
| **Sarrafzadegan et al., 2011** | JBI Cohort Studies | Adequate community-based cohort sampling in central Iran; regional rather than national. | Adequate: incident IHD was prospectively identified and adjudicated using standardized procedures. | Adequate follow-up and event surveillance for the reported incidence analysis. | Adequate person-time denominator and incidence estimation. | No major methodological concern within the source population; limited national generalizability. |
| **Talaei et al., 2013** | JBI Cohort Studies | Adequate population-based Isfahan Cohort Study sample; regional coverage. | Adequate: AMI/UAP/SCD outcomes adjudicated using medical records, death certificates, and verbal autopsy. | Adequate prospective follow-up/event ascertainment. | Adequate person-time denominators; coronary outcomes reported separately. | No major methodological concern within the source population. |
| **Khalili et al., 2014** | JBI Cohort Studies | Adequate population-based TLGS cohort; Tehran only. | Adequate prospective CHD outcome surveillance/adjudication. | Adequate long-term follow-up for incidence estimation. | Adequate person-time analysis; sex-specific estimates interpretable. | No major methodological concern within the Tehran population. |
| **Abbasi et al., 2018** | JBI Prevalence Studies | Adequate nationally representative SuRFNCD-2011 survey sampling. | Some concern: CHD was self-reported and stable angina was assessed separately. | Adequate survey coverage for national estimates. | Adequate weighted prevalence estimation; self-reported CHD and angina must remain distinct. | Some methodological concerns due principally to self-report/misclassification. |
| **Maleki et al., 2019** | JBI Prevalence Studies | Some concern: city-level population sample; limited external coverage. | Some concern: questionnaire/ECG/history-based CAD ascertainment and internal abstract/table inconsistency. | Adequate for cross-sectional survey, but reporting inconsistency reduces confidence. | Some concern: numerical inconsistency requires use of the clearly reported table/definition. | Some methodological concerns; retain with cautious interpretation. |
| **Ghaemian et al., 2020** | JBI Prevalence Studies | Adequate large population-based Tabari Cohort baseline sample; regional age range 35–70 y. | Some concern: CHD based on self-report. | Adequate baseline coverage. | Adequate prevalence analysis for the sampled population. | Some methodological concerns due to self-report and restricted regional/age coverage. |
| **Moazzeni et al., 2020** | JBI Prevalence Studies | Adequate population-based TLGS sample; Tehran only. | Adequate: CHD based on hospital records adjudicated by an outcome committee. | Adequate for cross-sectional prevalence analysis. | Adequate age-standardized prevalence estimation. | No major methodological concern within the Tehran population. |
| **Adib et al., 2020** | JBI Prevalence Studies | Adequate large Pars Cohort baseline sample; adults 40–75 y in Fars Province. | Some concern: IHD based on self-report. | Adequate baseline coverage. | Adequate prevalence calculation for the defined cohort. | Some methodological concerns due to self-report and restricted age/geographic coverage. |
| **Ahmadi et al., 2021** | JBI Prevalence Studies | Adequate large PERSIAN cohort sample including urban and rural participants; one province. | Some concern: physician-diagnosed IHD/MI history was self-reported. | Adequate baseline coverage. | Adequate prevalence analysis; IHD and MI kept separate. | Some methodological concerns due to self-report and subnational coverage. |
| **Sadabadi et al., 2022** | JBI Cohort Studies | Adequate prospective regional cohort framework; not nationally representative. | Adequate incident CHD ascertainment as reported; some detail on denominator/event definition is less explicit than in stronger cohorts. | Some concern: follow-up reporting is less complete for appraisal than in the major national/regional cohorts. | Some concern: precision/denominator reporting is limited for some sex-specific estimates. | Some methodological concerns; useful regional incidence evidence. |
| **Sarebanhassanabadi et al., 2024** | JBI Cohort Studies | Adequate community-based Yazd cohort; regional population. | Adequate clinical CAD outcome ascertainment during follow-up. | Some concern: approximately 20% of the recruited cohort was not included in the final analysis. | Adequate cumulative-incidence reporting; attrition may influence estimates. | Some methodological concerns, principally attrition. |
| **Farshidi et al., 2025** | JBI Prevalence Studies | Adequate multistage population sampling in Hormozgan; subnational rather than national. | Adequate: reported IHD was checked against medical records and confirmed by a cardiologist. | Adequate survey coverage for the target province. | Adequate prevalence analysis and subgroup reporting. | No major methodological concern within Hormozgan; limited national generalizability. |
| **Sabbagh Alvani et al., 2026** | JBI Prevalence Studies | Adequate nationally representative STEPS 2021 sample, restricted to adults ≥40 y for this analysis. | Some concern: lifetime CHD was based primarily on self-reported professional diagnosis/procedural history. | Adequate weighted survey coverage. | Adequate weighted prevalence estimation for adults ≥40 y. | Some methodological concerns due to self-report; age restriction defines, rather than invalidates, the target population. |
| **Registry/hospital surveillance and population mortality studies** | | | | | | |
| **Sarrafzadegan et al., 2009** | JBI Case Series + prespecified surveillance domains | Adequate multicenter surveillance across 13 Isfahan hospitals; captures hospitalized events rather than all population events. | Adequate standardized hospitalized MI ascertainment with 28-day follow-up. | Adequate for hospital surveillance; out-of-hospital events are outside the frame. | Some concern if interpreted as population incidence; appropriate as admission rate/CFR. | Some methodological concerns for population inference; strong regional hospital-surveillance evidence. |
| **Mohammadian-Hafshejani et al., 2014** | JBI Case Series + prespecified surveillance domains | Adequate 13-hospital surveillance among residents ≥65 y; age-restricted. | Adequate first-AMI ascertainment and 28-day follow-up. | Adequate within hospitalized elderly patients. | Adequate for admission/mortality patterns in the defined elderly population. | Some methodological concerns for generalization beyond elderly hospitalized patients. |
| **Mohammadian et al., 2015** | JBI Case Series + prespecified surveillance domains | Adequate long-running Isfahan AMI surveillance network; hospitalized events only. | Adequate first-AMI and 28-day mortality ascertainment. | Adequate trend follow-up within the surveillance system. | Adequate for 28-day CFR trends; overlaps the same surveillance network as related reports. | Some methodological concerns for population inference; useful temporal CFR evidence. |
| **Ahmadi et al., 2015 (EMHJ)** | JBI Case Series + prespecified surveillance domains | Broad national hospital registry covering all provinces; pre-hospital MI and nonparticipating capture may be missed. | Adequate ICD-defined new MI registry ascertainment. | Some concern: registry completeness outside participating hospitals/pre-hospital events cannot be assured. | Some concern: population denominators produce registry-based incidence, not complete population incidence. | Some methodological concerns; valuable national registry incidence estimate when labeled as registry-based. |
| **Ahmadi et al., 2015 (J Res Med Sci)** | JBI Case Series + prespecified surveillance domains | Same nationwide MI registry; provincial spatial coverage is broad. | Adequate registry MI ascertainment. | Some concern: spatial patterns depend on registry completeness and referral/case capture. | Adequate spatial methods for registry events; ecological interpretation required. | Some methodological concerns; supports geographic heterogeneity in hospitalized/registered MI. |
| **Ahmadi et al., 2015 (Int Cardiovasc Res J)** | JBI Case Series | Nationwide MI registry; limited to hospitalized cases. | Adequate for the crude in-hospital CFR; source-reported person-time mortality rates were not synthesized because the person-time units could not be verified. | Adequate for in-hospital case fatality; pre-hospital deaths excluded by design. | Some concern: rate/proportion terminology must be kept distinct. | Some methodological concerns; valid for in-hospital CFR, not population mortality. |
| **Ahmadi et al., 2015 (J Mazandaran Univ Med Sci)** | JBI Case Series + prespecified surveillance domains | Nationwide MI registry across 31 provinces; hospital deaths only. | Adequate confirmed MI and in-hospital death ascertainment. | Some concern: excludes out-of-hospital deaths; authors explicitly acknowledge this. | Major concern if population denominators are interpreted as total MI mortality; acceptable as geographic in-hospital death rate. | Some methodological concerns overall; major limitation for population-mortality interpretation. |
| **Namayande et al., 2016** | JBI Case Series + prespecified surveillance domains | Three main Yazd hospitals; completeness of population case capture uncertain. | AMI admission ascertainment is clinically based. | Some concern: hospital-only capture and modest number of records. | Major concern: reported overall incidence is internally inconsistent with sex-specific rates/denominators. | Major concern for the reported incidence rate; retain only with explicit caution. |
| **Nouri et al., 2020** | JBI Case Series + prespecified surveillance domains | Large multicenter Isfahan registry with long temporal coverage; hospitalized IHD only. | Adequate ICD and WHO-MONICA classifications. | Adequate within the hospital network; population event capture incomplete by design. | Some concern: hospitalization-derived rates are described as incidence and should not be interpreted as true population incidence. | Some methodological concerns; strong admission-trend evidence, not direct population incidence. |
| **Saadatagah et al., 2020** | JBI Case Series | Large single tertiary-center STEMI series over 12 years. | Adequate STEMI and in-hospital outcome ascertainment. | Adequate within the center; referral selection limits external validity. | Adequate for temporal in-hospital mortality/length-of-stay trends. | Some methodological concerns due to tertiary-center selection; useful hospital outcome trend. |
| **Mozaffarian et al., 2021** | JBI Case Series / prognostic follow-up | National first-MI registry cohort; includes patients reaching registry hospitals. | Adequate first-MI definition and post-event survival follow-up. | Some concern: pre-hospital deaths are not represented; follow-up pertains to registered MI survivors/patients. | Adequate for post-MI survival at reported time points. | Some methodological concerns for population fatality; useful national post-MI survival evidence. |
| **Soleimani & Bagheri, 2021** | JBI Case Series + prespecified surveillance domains | Nine Zanjan hospitals with provincial denominators; completeness of hospital capture uncertain. | Hospital-registered MI ascertainment. | Some concern: unregistered/out-of-hospital events may be missed. | Some concern: population incidence estimates depend on completeness of hospital capture. | Some methodological concerns; interpret as hospital-derived provincial incidence/spatial evidence. |
| **Ayat et al., 2022** | JBI Quasi-Experimental Studies | Two-city historical comparison of PEN-exposed and control populations; nonrandomized allocation. | Adequate ICD-defined hospital MI ascertainment. | Some concern: secular changes and differential case capture may influence before-after comparisons. | Some concern: difference-in-differences reduces but does not eliminate confounding. | Some methodological concerns; policy-effect estimates should be interpreted cautiously. |
| **Bahrani et al., 2023** | JBI Case Series | Large 13-hospital ACS surveillance registry over 15 years; regional hospital network. | Adequate ACS phenotyping and short-term mortality ascertainment. | Adequate within surveillance network; overlaps earlier Isfahan reports. | Adequate for phenotype-specific hospital CFR; not population mortality. | Some methodological concerns for population inference; strong hospital-surveillance evidence. |
| **Pourasghari et al., 2023** | JBI Cohort Studies (retrospective comparative) | Multicenter Tehran hospital sample comparing 2019 and 2020; not population based. | Adequate STEMI/NSTEMI hospital diagnosis. | Some concern: pandemic altered healthcare-seeking and admission thresholds. | Some concern: crude period comparison is vulnerable to case-mix and healthcare-use confounding. | Some methodological concerns; appropriate for pandemic-era hospital utilization/outcomes. |
| **Paknahad et al., 2024** | JBI Case Series | Very large 13-hospital Isfahan AMI registry over 2000–2017. | Adequate AMI ascertainment and 28-day follow-up within the registry. | Adequate long-term surveillance; retrospective registry dependence and overlap noted. | Adequate for temporal 28-day mortality trend; not population mortality. | Some methodological concerns for population inference; strong temporal registry evidence. |
| **Omranikhoo et al., 2013** | Prespecified population mortality-registry domains | Provincial death-registration dataset; rural/urban coverage reported. | Cause-specific IHD derived from registered causes of death. | Some concern: completeness and cause coding may vary over time/setting. | Adequate trend methods for registered mortality; validity depends on registration completeness. | Some methodological concerns typical of historical death-registration data. |
| **Mehrabani-Zeinabad et al., 2023** | JBI Quasi-Experimental Studies + prespecified mortality-registry domains | Population mortality comparison of intervention and control areas using DRS/cemetery/census sources. | IHD cause-specific mortality with explicit adjustment for incompleteness/misclassification. | Adequate use of multiple mortality sources; residual registration/coding differences remain possible. | Some concern: ecological intervention-control comparison cannot fully isolate the IHHP effect from secular confounding. | Some methodological concerns; strong corrected mortality-trend evidence, cautious causal interpretation. |
| **Sharifi et al., 2024** | Prespecified population mortality-registry domains | Population-based Fars death-registration study over 2004–2019. | ICD-coded IHD deaths; YLL calculated using a standard framework. | Some concern: cause-of-death misclassification/completeness may affect estimates. | Adequate mortality/YLL calculations; crude sex-specific rates should not be confused with ASRs. | Some methodological concerns; direct subnational mortality/YLL evidence. |
| **Khaleghi et al., 2026** | Prespecified population mortality-registry domains | Health authority and Civil Registration mortality data covering Mazandaran, 2011–2023. | ICD-10 I20–I25 cause-specific mortality. | Some concern: death-certificate misclassification and temporal registration quality. | Adequate joinpoint/trend approach if standardization is interpreted using the stated reference population. | Some methodological concerns; valuable recent population mortality trend evidence. |
| **Kazemy & Sharifzadeh, 2006** | JBI Case Series + prespecified surveillance domains | Hospital-based retrospective series from Birjand; regional hospital capture only | Definite hospitalized AMI identified from hospital records | Long 10-year observation period, but completeness of capture outside participating hospitals cannot be established | Descriptive annual counts only; no population denominator or standardized rates | Major concern for population inference. Useful only as a hospital-volume temporal trend and not as population incidence |
| **Model-based and burden-of-disease studies** | | | | | | |
| **Naghavi et al., 2009** | GATHER-informed health-estimate appraisal | Historical national NBD inputs; source-data completeness varies by indicator/province. | IHD mortality and DALY estimation based on NBD/WHO methods. | Some concern: historical data availability and adjustment assumptions are incompletely transparent by current standards. | Uncertainty/model dependence less fully reported than in modern GBD publications. | Some methodological/reporting concerns; important historical national benchmark. |
| **Maracy et al., 2015** | GATHER-informed health-estimate appraisal | GBD 2010 model inputs and national estimates; derived from a common GBD framework. | Standardized GBD cause and DALY estimation. | Adequate reporting of key trends, but dependent on vintage-specific GBD inputs/model assumptions. | Uncertainty intervals reported; reproducibility is constrained by model dependence. | Some methodological/reporting concerns inherent to model-based GBD estimates. |
| **Shams-Beyranvand et al., 2017** | GATHER-informed health-estimate appraisal | Regional Isfahan inputs combining death registration and prevalence information. | IHD DALY/YLL/YLD with explicit incompleteness/misclassification adjustment. | Adequate description of correction approach; results depend on input completeness and disability assumptions. | Uncertainty intervals reported for key rates; model assumptions remain influential. | Some methodological/reporting concerns; useful corrected regional burden estimate. |
| **Ebrahimi et al., 2017** | GATHER-informed health-estimate appraisal | Regional MI event/death inputs from West Azerbaijan/Urmia. | MI DALY/YLL/YLD calculation using standard burden concepts. | Some concern: completeness of event/death capture and documentation of input-data limitations. | Burden calculations are interpretable, but uncertainty/model sensitivity is less extensively characterized. | Some methodological/reporting concerns. |
| **Kiani et al., 2019** | GATHER-informed health-estimate appraisal | Isfahan mortality inputs used for age-period-cohort/MIAMOD estimation and projection. | Observed data combined with model-based mortality estimation/projection. | Some concern: projection validity depends strongly on structural assumptions and future trend stability. | Model-based uncertainty/assumption dependence is central to projected estimates. | Major concern for projected estimates; observed historical trends are more reliable than forecasts. |
| **Moradi et al., 2021** | GATHER-informed health-estimate appraisal | Kurdistan regional mortality/morbidity inputs over 2011–2017. | WHO-style DALY estimation including IHD components. | Some concern: completeness of regional source data and IHD-specific input documentation. | Burden calculations are transparent at summary level; uncertainty reporting is limited relative to GBD. | Some methodological/reporting concerns. |
| **Naderimagham et al., 2022** | GATHER-informed health-estimate appraisal | National/provincial death-registration inputs with correction for incompleteness and misclassification. | Modeled IHD mortality using redistribution/correction and spatiotemporal methods. | Adequate description of correction/modeling framework; estimates remain model dependent. | Uncertainty and model-based results reported; interpretation should distinguish observed from estimated values. | Some methodological/reporting concerns; comparatively strong model-based mortality analysis. |
| **Koolaji et al., 2023** | GATHER-informed health-estimate appraisal | GBD 2019 Iran-specific national and provincial estimates derived from shared GBD inputs. | Standard GBD incidence, prevalence, mortality, DALY/YLL/YLD estimation. | Adequate reporting of major inputs/results at publication level; full reproducibility depends on GBD infrastructure. | 95% uncertainty intervals reported; release-specific model assumptions apply. | Some methodological/reporting concerns inherent to modeled estimates; primary GBD 2019 Iran report. |
| **Mousavi et al., 2023** | GATHER-informed health-estimate appraisal | GBD 2019 estimates restricted to adults ≥70 y; same underlying model vintage as other GBD 2019 reports. | Standard GBD IHD burden estimation. | Adequate age-specific reporting; not an independent data source from GBD 2019. | Uncertainty reported; overlap must be handled at synthesis level. | Some methodological/reporting concerns; useful age-specific derivative analysis. |
| **Karamitanha et al., 2024** | GATHER-informed health-estimate appraisal | GBD 2019 provincial estimates; same model vintage/input family as Koolaji and other GBD 2019 reports. | Standard GBD provincial IHD incidence/mortality estimation. | Adequate subnational reporting; model/source overlap is substantial. | Uncertainty/model dependence should be interpreted within GBD 2019, not as independent replication. | Some methodological/reporting concerns; useful provincial derivative analysis. |
| **Heidari-Foroozan et al., 2025** | GATHER-informed health-estimate appraisal | GBD 2021 national/subnational estimates. | Standard GBD IHD mortality/DALY estimation; article narrative appears to transpose nonfatal incidence/prevalence values. | Adequate for mortality/DALY reporting; major internal reporting concern for exact nonfatal values. | Uncertainty intervals reported, but nonfatal values should be verified against original GBD results before use. | Some concerns overall; major concern for nonfatal incidence/prevalence values in the article narrative. |
| **Sepanlou et al., 2026** | GATHER-informed health-estimate appraisal | GBD 2023 national and provincial cause-of-death estimates. | Standard GBD cause-specific mortality estimation; this report does not provide national IHD nonfatal estimates. | Adequate reporting of mortality trends/subnational estimates; release-specific model assumptions apply. | 95% uncertainty intervals reported; cross-vintage comparisons require caution. | Some methodological/reporting concerns inherent to modeled mortality estimates; strong source for GBD 2023 IHD mortality. |
| **Aminorroaya et al., 2024** | GATHER-informed health-estimate appraisal | health-estimate appraisal  GBD 2019 estimates for the 21 NAME countries, including Iran; same underlying GBD 2019 model/data family as Koolaji, Mousavi, and Karamitanha | Standard GBD IHD framework; incidence, prevalence, mortality, YLL, YLD, DALY, premature mortality, and attributable risks; demographic decomposition of incident cases | Adequate publication-level transparency; GBD data sources and GHDx/GBD Compare identified, GATHER compliance stated, and decomposition methods described; completeness of individual Iran-specific source inputs cannot be independently assessed from the report | Counts and age-standardized rates with 95% UI; sex- and country-specific comparisons and decomposition analysis appropriately reported; estimates remain model-dependent and release-specific | Some methodological/reporting concerns. Useful complementary GBD 2019 derivative for premature mortality, NAME comparisons, and demographic decomposition, but not independent evidence from other GBD 2019 reports |

**Notes:** Methodological appraisal was performed independently by two reviewers, with disagreements resolved by consensus and consultation with a third reviewer when necessary. For registry and hospital-surveillance studies, the applicable JBI checklist was supplemented by prespecified surveillance domains addressing source-population/catchment coverage, case ascertainment, completeness of event capture or follow-up, denominator validity, and temporal or geographic consistency. For population mortality studies, prespecified domains included completeness of death registration, cause-of-death classification, denominator validity, standardization, and temporal consistency. GATHER-informed domains for model-based studies addressed transparency of input data, estimation methods, uncertainty, and model dependence. “No major methodological concern,” “Some methodological concerns,” and “Major concern for specific estimate” are review-level summary judgments and are not JBI- or GATHER-defined categories or numerical scores. These judgments were used to inform interpretation rather than eligibility. JBI = Joanna Briggs Institute; GATHER = Guidelines for Accurate and Transparent Health Estimates Reporting; GBD = Global Burden of Disease; MI = myocardial infarction; IHD = ischemic heart disease; CFR = case-fatality rate; DRS = death registration system; DALY = disability-adjusted life year; YLL = years of life lost; YLD = years lived with disability.

# Table S7. Detailed incidence estimates for ischemic heart disease and myocardial infarction in Iran.

| **Study/source** | **Period/setting** | **Design/data source** | **Outcome/definition** | **Principal incidence estimate** | **Sex/geographic detail** | **Interpretive note** |
| --- | --- | --- | --- | --- | --- | --- |
| **Panel A. Empirical population-based or registry-derived incidence estimates** | | | | | | |
| Sarrafzadegan et al., 2011 | ICS; baseline 2001; median follow-up 4.8 y | Prospective population cohort; 24,379 PY | Incident IHD; adjudicated | 219 events; 8.9 (7.8–10.2)/1,000 PY | Men 10.6 (8.8–12.5); women 7.4 (6.0–9.0)/1,000 PY | Population-based regional incidence. |
| Talaei et al., 2013 | ICS follow-up; central Iran | Prospective cohort; 32,893 PY | AMI, UAP, SCD; adjudicated | AMI: 352 men vs 186 women/100,000 PY | UAP: 352 men vs 584 women; SCD: 220 men vs 104 women/100,000 PY | Individual coronary outcomes kept separate. |
| Khalili et al., 2014 | 1999–2010; TLGS | Prospective population cohort | Incident CHD | Crude 11.9 (10.6–13.2) men vs 6.5 (5.7–7.3) women/1,000 PY | Iran-standardized 10.5 vs 6.1; WHO-standardized 12.2 vs 7.4/1,000 PY | Sex-specific Tehran incidence. |
| Ahmadi et al., 2015 (EMHJ) | 2012; Iranian MI Registry | Nationwide hospital registry; 20,750 new MI cases | AMI, ICD-10 I21–I22 | Age-standardized 73.3/100,000 (72.3–74.3); crude 64.9/100,000 | Male:female incidence ratio 2.63; province range 24.5 Qom to 152.5 North Khorasan | Registry-derived incidence; pre-hospital MI deaths not captured. |
| Namayande et al., 2016 | 2013; three main Yazd hospitals | Hospital-admission GIS study; 440 records | AMI hospital admissions | Reported overall rate 783/100,000 PY | Men 531; women 458/100,000 PY | Overall rate is internally unusual relative to sex-specific rates. |
| Soleimani & Bagheri, 2021 | 2014–2018; nine Zanjan hospitals | Retrospective geospatial registry; n=3,820 | Hospital-registered MI | Overall age-adjusted 343/100,000; annual 88→114/100,000 PY | Approx. men 470 vs women 204/100,000; Golabar 515/100,000 | Hospital capture with population denominators. |
| Ayat et al., 2022 | 2015 vs 2019; Shahreza vs Najafabad | Historical cohort from hospital information systems | Definite MI, ICD-10 I21.0–I21.9 | Shahreza 1.85→2.43/1,000; control 2.46→3.35/1,000 | Difference-in-differences −0.31/1,000 | Policy evaluation; hospital-ascertained events. |
| Sadabadi et al., 2022 | MASHAD; 6-y follow-up | Prospective population cohort | Incident CHD | Men 19.20 (8.10–30.30)/1,000 PY | Women 11.60 (7.30–15.90)/1,000 PY | Regional prospective incidence. |
| Sarebanhassanabadi et al., 2024 | Yazd Healthy Heart Project; mean follow-up 9.9 y | Community cohort; n=1,552; 15,420.11 PY | Incident clinical CAD | 225 events; cumulative incidence 14.5% (12.4–16.6) | Incidence 16.8 (14.4–19.2) men vs 12.0 (9.5–14.5) women/1,000 PY | ~20% attrition from 2,000 recruited. |
| **Panel B. Model-based incidence estimates** | | | | | | |
| Koolaji et al., 2023 (GBD 2019) | 1990–2019; Iran | GBD 2019 | IHD | ASIR 898.3 (784.0–1,026.5) → 829.1 (719.9–945.2)/100,000 | 2019 men 1,007.8; women 652.0; cases 218,181→593,001 | Primary Iran-specific GBD 2019 analysis. |
| Karamitanha et al., 2024 (GBD 2019) | 1990–2019; 31 provinces | GBD 2019 provincial analysis | IHD | National trend derived from GBD 2019; incidence decreased ~6% in women and ~4% in men | 2019 highest incidence North Khorasan; Khorasan Razavi ~−13%; Ardabil ~−1% | Derivative of same GBD 2019 model source. |
| Heidari-Foroozan et al., 2025 (GBD 2021) | 1990–2021; Iran | GBD 2021 | IHD | Not tabulated: article appears to transpose incidence and prevalence labels/values | Source verification required | Mortality/DALY retained; nonfatal values withheld. |

Rates are reproduced in the units used by the original reports. Hospital-derived rates were not treated as equivalent to incidence measured in prospective population cohorts. GBD estimates use 95% UI; empirical studies generally use 95% CI.

# Table S8. Detailed prevalence estimates for ischemic heart disease, coronary heart disease, coronary artery disease, and myocardial infarction in Iran.

| **Study/source** | **Setting/population** | **Data source/ascertainment** | **Phenotype** | **Overall prevalence** | **Sex/age/geographic detail** | **Interpretive note** |
| --- | --- | --- | --- | --- | --- | --- |
| **Panel A. Empirical prevalence estimates** | | | | | | |
| Ghanbarian et al., 2004 | TLGS baseline; n=5,539 adults ≥30 y | Minnesota-coded resting ECG | Possible/probable MI | 1.2% | Men 1.8%; women 0.8% | Population-based Tehran; ECG-defined MI. |
| Sadeghi et al., 2006 | IHHP baseline; n=6,498 >35 y | Rose questionnaire + Minnesota ECG | CAD | NR overall | Women 37.5%; men 22.2% | Broad symptom/ECG definition. |
| Nabipour et al., 2006 | Persian Gulf Healthy Heart Study; n=3,723 ≥25 y | Minnesota-coded ECG | Nonfatal IHD | 12.7% | Men 10.4%; women 14.7% | Regional ECG-defined IHD. |
| Fakhrzadeh et al., 2008 | Qazvin; n=846 adults ≥25 y | Clinical survey + ECG | Possible MI / ischemic ECG / angina | Possible MI 4.2%; ischemic ECG changes 36.8%; angina 2.2% | Age-adjusted | Distinct outcomes; do not sum. |
| Hadaegh et al., 2009 | TLGS baseline; n=5,984 adults ≥30 y | Rose angina + self-report + Minnesota ECG | CHD | 21.8% age-adjusted | Women 22.3%; men 18.8% | Broad composite CHD definition. |
| Abbasi et al., 2018 | SuRFNCD-2011; national; n=11,867 | Standardized survey; self-report | CHD / stable angina | CHD 5.3% (4.6–5.9); stable angina 7.7% (4.6–8.7) | CHD urban 5.6%, rural 4.3%; stable angina urban 7.9%, rural 7.4% | Nationally representative; self-report. |
| Maleki et al., 2019 | Borujerd; n=801 adults >35 y | Rose + Minnesota ECG + documented CAD history | Probable/definite CAD | Probable 18.7%; definite 15.7% | Rose probable 12.1%, definite 7.5%; Minnesota probable 14.4%, definite 9.1%; documented definite 9.1% | Final diagnostic table values used; abstract internally inconsistent. |
| Ghaemian et al., 2020 | Tabari Cohort; n=10,255, age 35–70 y | Self-reported history | CHD | 9.2% | Rural 12.1% vs urban 7.9% | Regional cohort baseline; self-report. |
| Moazzeni et al., 2020 | TLGS 2008–2011; n=7,718 ≥30 y | Hospital records adjudicated by outcome committee | CHD | 7.71% (7.18–8.24), age-standardized | Men 8.62%; women 7.19% | Population-based Tehran estimate with adjudication. |
| Adib et al., 2020 | Pars Cohort; n=9,248 age 40–75 y | Self-reported history | IHD | 10.2% (9.5–10.9) | NR | Regional cohort baseline. |
| Ahmadi et al., 2021 | Shahrekord PERSIAN; n=10,075 age 35–70 y | Self-reported physician diagnosis | IHD / MI | IHD 5.7%; MI 1.3% | IHD men 6.9%, women 4.7%; urban 6.3%, rural 4.4% | IHD and MI kept separate. |
| Farshidi et al., 2025 | Hormozgan; n=6,289 adults ≥20 y | Self-report + medical record/cardiologist confirmation | IHD | 6.7% | Men 7.5%, women 6.1%; urban 6.9%, rural 6.5% | Recent subnational population survey. |
| Sabbagh Alvani et al., 2026 | Iran STEPS 2021; n=16,552 adults ≥40 y | STEPS self-report of physician diagnosis/revascularization | CHD | 10.43% (9.91–10.96) | Women 8.39%, men 12.92%; 40–59 y 5.66%, ≥60 y 19.09%; urban 10.86%, rural 9.03% | Nationally representative for ≥40 y; self-reported lifetime CHD. |
| **Panel B. Model-based or burden-study prevalence estimates** | | | | | | |
| Ebrahimi et al., 2017 | West Azerbaijan, 2012–2013 | Regional MI burden study | MI | 376/100,000 | NR | Burden-study prevalence rate; not national. |
| Koolaji et al., 2023 (GBD 2019) | 1990–2019; Iran | GBD 2019 | IHD | 6,250.6 (5,678.7–6,852.2) → 6,198.5 (5,644.4–6,814.6)/100,000 | 2019 men 7,597.1; women 4,815.8; prevalent cases 1.49→4.34 million | Modeled age-standardized prevalence. |
| Mousavi et al., 2023 (GBD 2019; age ≥70) | 2019; Iran adults ≥70 y | GBD 2019 age-specific analysis | IHD | 52,886.4 (47,543.0–58,381.6)/100,000 | 1.836 million prevalent cases in 2019; ~7.5% higher rate than 1990 | Age-restricted; overlaps GBD 2019. |
| Heidari-Foroozan et al., 2025 (GBD 2021) | 1990–2021; Iran | GBD 2021 | IHD | Not extracted because incidence/prevalence labels/values appear transposed | Source verification required | Nonfatal estimates withheld. |

Prevalence estimates retain the original study-specific disease definitions. ECG-defined disease, questionnaire-defined angina/CHD, physician-diagnosed self-report, and model-based GBD prevalence were not treated as interchangeable.

# Table S9. Hospitalization and acute coronary surveillance estimates.

| **Study/report** | **Period/setting** | **Data source/population** | **Phenotype** | **Admission/incidence estimate** | **Mortality/CFR/survival** | **Interpretive note** |
| --- | --- | --- | --- | --- | --- | --- |
| Sarrafzadegan et al., 2009 | 2000–2004; 13 Isfahan hospitals | WHO-MONICA-adapted surveillance | Hospitalized MI | Age-adjusted admission rate 131.67→209.27/100,000 | 28-day CFR women 23.1%, men 13.2% | Admission rate is not population incidence. |
| Mohammadian-Hafshejani et al., 2014 | 2002–2007; 13 Isfahan hospitals | First AMI in residents ≥65 y; 3,990 analyzed | First AMI, ICD-10 | Seasonal variation in admissions | 724/3,990 (18.1%) died by 28 d | Elderly-only surveillance. |
| Mohammadian et al., 2015 | 2000–2009; Isfahan AMI surveillance | First AMI; n=12,900 | First AMI | NR | 28-day CFR 11.2%→7.9%; men 9.2%→6.7%; women 16.1%→10.9% | ~29.5% overall CFR reduction; overlapping surveillance source. |
| Ahmadi et al., 2015 (EMHJ) | 2012; Iranian MI Registry | Nationwide hospital registry; 20,750 new hospital-registered MI cases. | AMI, ICD-10 I21–I22 | Age-standardized registry incidence 73.3/100,000 | NR in incidence report | National registry; misses MI not reaching registered hospitals. |
| Ahmadi et al., 2015 (Int Cardiovasc Res J) | Apr 2012–Mar 2013; Iranian MI Registry | Prospective national hospital cohort; n=20,750 | Confirmed acute MI | NR | 2,511 in-hospital deaths among 20,750 MI admissions; crude in-hospital CFR 12.1%. | In-hospital outcome only; pre-hospital deaths were not captured. Source-reported person-time mortality rates were not synthesized because the reported person-time units could not be verified. |
| Ahmadi et al., 2015 (J Res Med Sci) | 2012; Iranian MI Registry | National spatial analysis; n=20,750 | New MI cases | High-incidence clusters: North Khorasan, Yazd, Kerman, Semnan, Golestan, Mazandaran | NR | Same registry source; geographic heterogeneity. |
| Ahmadi et al., 2015 (J Mazandaran Univ Med Sci) | 2012/2013; Iranian MI Registry | Prospective hospital-based national registry; n=20,750 | MI in-hospital death; spatial analysis | Highest standardized in-hospital mortality incidence/100,000: Ardabil 31; North Khorasan 23.7; Yazd 20.2; West Azerbaijan 19.1; Khuzestan 18.4 | 2,511 deaths (12.1%); women CFR 14.96%, men 11.02%; global Moran I 0.141, p=0.126 | Population denominators were used for geographic rates, but only in-hospital deaths were captured. |
| Namayande et al., 2016 | 2013; three Yazd hospitals | Hospital-admission GIS; 440 records | AMI admissions | Reported overall 783/100,000 PY; men 531, women 458 | NR | Internal numerical inconsistency warrants caution. |
| Nouri et al., 2020 | 2001–2016; Isfahan hospitals | Multicenter registry; 102,254 IHD hospitalizations; 48,465 WHO-MONICA | IHD | Adjusted hospitalization-derived rates ~27–37/10,000 in 2001–02 to ~39–51/10,000 in 2015–16; annual increase 1.42%–3.36% | NR | Hospitalization-derived, not true population incidence. |
| Saadatagah et al., 2020 | 2006–2017; Tehran Heart Center | Single-center STEMI database; n=8,295 | STEMI | NR | In-hospital mortality 8.0%→3.9%; stay 8.4→5.2 d | Longitudinal hospital trend. |
| Mozaffarian et al., 2021 | National MI Registry; n=21,181 first MI | Follow-up to 1 y | First MI | NR | 2,479 deaths (11.7%); survival 95% at 28 d, 90% at 6 mo, 88% at 1 y | Excludes pre-hospital deaths. |
| Soleimani & Bagheri, 2021 | 2014–2018; nine Zanjan hospitals | Retrospective geospatial registry; n=3,820 | MI | Age-adjusted 343/100,000; annual 88→114/100,000 PY | NR | Hospital capture with population denominator. |
| Ayat et al., 2022 | 2015 vs 2019; Shahreza vs Najafabad | Hospital information systems | Definite MI | Shahreza 1.85→2.43/1,000 vs control 2.46→3.35/1,000 | MI mortality difference-in-differences ~−9/100,000 | Policy evaluation; possible secular/confounding effects. |
| Bahrani et al., 2023 | 2001–2016; 13 Isfahan hospitals | Prospective ACS surveillance; n=62,276 | STEMI/NSTEMI/UAP | Large longitudinal ACS admission series | STEMI in-hospital 6.5%; 28-day 12.6% | Regional hospital surveillance. |
| Pourasghari et al., 2023 | 2019 vs 2020; Tehran | Multicenter hospital study; n=4,560 AMI admissions | STEMI/NSTEMI | Admissions −12.65% | In-hospital mortality 4.9%→7.0%; OR 1.42 (1.11–1.82) | Pandemic utilization/outcome comparison. |
| Paknahad et al., 2024 | 2000–2017; Isfahan registry | 13-hospital retrospective cohort; n=121,284 AMI | AMI; 28-d follow-up | NR | 28-day mortality declined from 2006 onward; ~60% lower in 2014–2017 vs 2000–2002 | Very large overlapping Isfahan surveillance source. |
| Kazemy & Sharifzadeh, 2006 | 1994–2003; Birjand hospitals | Retrospective hospital records; 918 AMI hospitalizations | Definite hospitalized AMI | Annual admissions increased from 47 to 201 | NR | Hospital-volume trend only; no population denominator |

# Table S10. Detailed mortality, case-fatality, and burden-of-disease estimates.

| **Study/source** | **Period/setting** | **Data source/outcome** | **Mortality/CFR estimate** | **DALY/YLL/YLD or related burden** | **Interpretive note** |
| --- | --- | --- | --- | --- | --- |
| **National/subnational population mortality and burden studies** | | | | | |
| Naghavi et al., 2009 (Iran NBD 2003) | 2003; national | National Burden of Disease model | IHD mortality 109/100,000 | YLL 771/100,000; YLD 539/100,000; DALY 1,310/100,000; total 871,336 DALYs | Older NBD methodology. |
| Maracy et al., 2015 (GBD 2010) | 1990–2010; Iran | GBD 2010 | AS mortality 222 (207–243) →175 (152–190)/100,000 | Men 264→218; women 178→130; DALY ASR 4,720→3,245; deaths 52,874→89,250 | Absolute deaths rose despite declining AS mortality. |
| Shams-Beyranvand et al., 2017 | 2014; Isfahan | Regional burden model | NR as principal headline | DALYs 43,517.71; YLL 31,891.79; YLD 11,625.92; DALY rate 3,953.31/100,000 | Subnational modeled burden. |
| Ebrahimi et al., 2017 | 2012–2013; West Azerbaijan | Regional MI burden | MI accounted for 18.8% of all deaths | DALYs 53,804 y; YLL 52,170; YLD 1,634; 7,235 MI occurrences | Regional MI burden. |
| Kiani et al., 2019 | Observed 2004–2013; projected 2014–2018; Isfahan | Age-period-cohort/MIAMOD mortality modeling | Observed average annual increase 13.6% men, 14.7% women; projected ~14.1% and 10% | NR | Projection-dependent; differs methodologically from other mortality series. |
| Moradi et al., 2021 | 2011–2017; Kurdistan | Regional CVD burden | NR IHD-specific mortality rate in principal summary | IHD 71,102.3 DALYs (33.8% of total CVD DALYs) | Subnational burden. |
| Naderimagham et al., 2022 | 1990–2015; national/provincial | Corrected DRS + spatiotemporal/Gaussian-process modeling | AS IHD mortality 49.0 (34.8–67.9) → peak 146.5 (112.0–187.4) → 91.6 (71.9–113.7)/100,000 | 2015 women 79.1, men 104.1; province range 58.0–136.2; deaths 41,885 men + 31,622 women | Corrected/model-based DRS estimate. |
| Omranikhoo et al., 2013 | 2004–2011; Bushehr | Population death registration | IHD mortality declined ~8% per year in rural and urban populations | NR | Avoidable-mortality framework. |
| Mehrabani-Zeinabad et al., 2023 | 2000–2018; Isfahan vs Arak | DRS/cemetery/census trend | Standardized IHD mortality fell 44.62% during 2002–2011, then increased after 2011 | NR | Avoid causal attribution to IHHP. |
| Sharifi et al., 2024 | 2004–2019; Fars | Population death registration | 46,969 IHD deaths; crude mortality 84.2/100,000 men, 66.5 women | YLL 287,625 men; 209,665 women | Direct subnational mortality/YLL evidence. |
| Khaleghi et al., 2026 | 2011–2023; Mazandaran | Health authority + Civil Registration | IHD 31,135 deaths (42.39% of CVD deaths) | Overall CVD ASMR 173.7→167.3/100,000; IHD-specific joinpoint trends in source | Recent population mortality through 2023. |
| **GBD 2019/2021/2023 mortality and burden estimates** | | | | | |
| Koolaji et al., 2023 (GBD 2019) | 1990–2019; Iran | GBD 2019 | AS mortality 285.7 (260.8–307.1) →163.6 (149.0–176.2)/100,000 | 2019 men 177.2, women 150.9; DALY ASR 5,437.6→2,842.7; YLL 5,356.1→2,761.7; YLD 81.5→81.0 | Primary Iran-specific GBD 2019 analysis. |
| Mousavi et al., 2023 (GBD 2019, age ≥70) | 1990–2019; adults ≥70 y | GBD 2019 age-specific analysis | 2019 deaths 67.1 thousand; mortality 1,932.9 (1,720.8–2,094.2)/100,000, ~22% lower than 1990 | DALYs 820.4 thousand; DALY rate 23,630.9/100,000, ~33.7% lower | Age-restricted; overlaps GBD 2019. |
| Karamitanha et al., 2024 (GBD 2019) | 1990–2019; 31 provinces | GBD 2019 provincial analysis | Mortality decreased ~35% in women and ~40% in men | 2019 lowest mortality Tehran; highest Golestan | Provincial derivative of GBD 2019. |
| Heidari-Foroozan et al., 2025 (GBD 2021) | 1990–2021; Iran | GBD 2021 | AS IHD mortality 259.88 (238.06–276.23) →146.11 (130.71–156.95)/100,000 | DALY ASR 5,166.82→2,731.30 | Nonfatal values withheld due article-level inconsistency. |
| Sepanlou et al., 2026 (GBD 2023) | 1990–2023; national + 31 provinces | GBD 2023 cause-of-death analysis | 2023 IHD AS mortality 127.2 (104.5–149.1)/100,000 | IHD mortality decline contributed 2.61 y to life-expectancy gain; provincial ASMR 88.1 Tehran to 262.8 Qom | Mortality-focused; no IHD incidence/prevalence in report. |

# Table S10. Detailed mortality, case-fatality, and burden-of-disease estimates (continued).

| **Study/source** | **Period/setting** | **Data source/outcome** | **Mortality/CFR estimate** | **DALY/YLL/YLD or related burden** | **Interpretive note** |
| --- | --- | --- | --- | --- | --- |
| **Registry/hospital case-fatality and survival estimates** | | | | | |
| Sarrafzadegan et al., 2009 | 2000–2004; Isfahan | MI surveillance; 28-day follow-up | 28-day CFR women 23.1%, men 13.2% | Admission rate 131.67→209.27/100,000 | Hospital surveillance. |
| Mohammadian-Hafshejani et al., 2014 | 2002–2007; Isfahan; age ≥65 y | First AMI; 28-day follow-up | 724/3,990 (18.1%) died by 28 d | Seasonal pattern evaluated | Elderly-only. |
| Mohammadian et al., 2015 | 2000–2009; Isfahan | First AMI; 28-day follow-up | 28-day CFR 11.2%→7.9%; women 16.1%→10.9%; men 9.2%→6.7% | ~29.5% overall reduction | Overlapping Isfahan surveillance. |
| Ahmadi et al., 2015 (Int Cardiovasc Res J) | 2012–2013; national MI registry | In-hospital mortality | 2,511 deaths among 20,750 admissions; crude in-hospital CFR 12.1%. | Sex-specific crude CFR not reported in this article. | In-hospital outcome only. |
| Ahmadi et al., 2015 (J Mazandaran Univ Med Sci) | 2012/2013; national MI registry | Geographic in-hospital mortality | 2,511 deaths (12.1%); women CFR 14.96%, men 11.02% | Highest standardized in-hospital mortality incidence/100,000: Ardabil 31; North Khorasan 23.7; Yazd 20.2; West Azerbaijan 19.1; Khuzestan 18.4; Moran I 0.141, p=0.126 | Only in-hospital deaths captured. |
| Saadatagah et al., 2020 | 2006–2017; Tehran | STEMI database | In-hospital mortality 8.0%→3.9% | Hospital stay 8.4→5.2 d | Single tertiary center. |
| Mozaffarian et al., 2021 | National MI Registry; first MI | Follow-up to 1 y | 2,479 deaths (11.7%); survival 95% at 28 d, 90% at 6 mo, 88% at 1 y | NR | Excludes pre-hospital deaths. |
| Bahrani et al., 2023 | 2001–2016; 13 Isfahan hospitals | ACS registry | STEMI in-hospital 6.5%; 28-day 12.6% | NR | Regional surveillance. |
| Pourasghari et al., 2023 | 2019 vs 2020; Tehran | AMI hospitalizations | In-hospital mortality 4.9%→7.0%; OR 1.42 (1.11–1.82) | Admissions −12.65% | Pandemic comparison. |
| Paknahad et al., 2024 | 2000–2017; Isfahan; n=121,284 | AMI 28-day follow-up | ~60% lower 28-day mortality in 2014–2017 vs 2000–2002 | Decline evident from 2006 | Overlapping Isfahan surveillance. |

Population mortality, modeled mortality, hospital case-fatality, incidence-density mortality, and post-MI survival are distinct outcomes. Successive GBD releases are separate model vintages.
